# Apelin conformational and binding equilibria upon micelle interaction primarily depend on membrane-mimetic headgroup

**DOI:** 10.1038/s41598-017-14784-0

**Published:** 2017-11-13

**Authors:** Kyungsoo Shin, Muzaddid Sarker, Shuya K. Huang, Jan K. Rainey

**Affiliations:** 1 Department of Biochemistry & Molecular Biology, Dalhousie University, Halifax, Nova Scotia B3H 4R2 Canada; 2 Department of Chemistry, Dalhousie University, Halifax, Nova Scotia B3H 4R2 Canada

## Abstract

Apelin is one of two peptide hormones that activate the apelin receptor (AR or APJ) to regulate the cardiovascular system, central nervous system, and adipoinsular axis. Here, we apply circular dichroism (CD) spectropolarimetry and nuclear magnetic resonance (NMR) spectroscopy to characterize the potential membrane binding by the two longest bioactive apelin isoforms, apelin-55 and -36, using membrane-mimetic dodecylphosphocholine (DPC), sodium dodecyl sulfate (SDS), and 1-palmitoyl-2-hydroxy-*sn*-glycero-3-[phospho-*rac*-(1-glycerol)] (LPPG) micelles. Pulsed field gradient diffusion NMR experiments demonstrated preferential interaction of both apelin-55 and -36 with anionic SDS and LPPG micelles over zwitterionic DPC micelles. Chemical shift perturbations and changes in ps-ns scale dynamics of apelin-55 in all micelles were similarly localized along the polypeptide backbone, demonstrating clear dependence upon detergent headgroup, while comparison of chemical shifts between apelin-55 and apelin-36 showed negligible differences indicative of highly similar modes of micelle interaction. Notably, the observed behaviour was consistent with an ensemble averaged pair of free and bound states in fast exchange on the NMR timescale proportional to the fraction of micelle-bound protein, implying a similar conformational equilibrium regardless of headgroup and tailgroup. Membrane catalysis of apelin-AR binding would thus give rise to analogous behaviour in the essential C-terminal region common to all apelin isoforms.

## Introduction

The apelin receptor (APJ/APLNR; abbreviated AR herein) is a class A G-protein coupled receptor for two peptide hormones, apelin and apela (ELABELA/Toddler)^1^. Activation of this receptor by apelin or apela has been implicated in a variety of physiological and pathological processes. For example, AR activation by apelin has been shown to modulate the cardiovascular system with a potent inotropic effect, to regulate energy metabolism and diabetes, and to alter tumour formation^1^. While apela can exert similar effects in many cases^2–5^, this ligand is most recognized for its essential roles in embryonic development^6,7^.

Apelin is initially produced as a 77-residue preprotein with a 22-residue signal peptide. Cleavage of this N-terminal signal peptide produces a 55-residue isoform, apelin-55, previously referred to as proapelin but recently found in body fluids^8^ and, subsequently, to activate the AR^9^. Apelin-55 can be further processed to shorter, N-terminally truncated isoforms by proteases such as proprotein convertase subtilisin kexin subtype 3 (PCSK3)^10^, with the C-terminal region being required for activation of the AR. The predominant isoforms identified *in vivo* are apelin-36, -17, -13, and pyroglutamate-apelin-13 (Pyr-apelin-13, having an N-terminal pyroglutamate residue)^11–14^, named according to the number of C-terminal residues retained (Table 1). Apela, on the other hand, is initially produced as a 54-residue preprotein with a 22-residue signal peptide^6^. Analogous to apelin, removal of the signal peptide also yields the longest bioactive isoform apela-32, which can be further N-terminally truncated to produce shorter isoforms of 22-, 21-, or 11-residues in length^4,6,7^ (Table 1).

**Table 1:** Amino acid sequences and physicochemical characteristics of prevalent apelin and apela isoforms.

Isoform	Amino acid sequence^*^	Net charge (pH 7)^#^	Hydrophobic residues^‡^
Apelin-55^†^	SGSLMPLPDGNGLEDGNVRHLVQPRGSRNGPGPWQGGRRKFRRQRPRLSHKGPMPF	8.2	12
Apelin-36	LVQPRGSRNGPGPWQGGRRKFRRQRPRLSHKGPMPF	10.1	6
Apelin-17	KFRRQRPRLSHKGPMPF	6.1	4
Apelin-13	QRPRLSHKGPMPF	3.1	3
Pyr-apelin-13	<ERPRLSHKGPMPF	3.1	3
Apela-32	QRPVNLTMRRKLRKHNCLQRRCMPLHSRVPFP	9.1	9
Pyr-apela-32	<ERPVNLTMRRKLRKHNCLQRRCMPLHSRVPFP	9.1	9
Apela-22	KLRKHNCLQRRCMPLHSRVPFP	6.1	6
Apela-21	LRKHNCLQRRCMPLHSRVPFP	5.1	6
Apela-11	CMPLHSRVPFP	1.0	4

^*^<E represents pyroglutamate.

^#^Net charge was determined using the equation Z = ${\sum }_{i}{N}_{i}\frac{{10}^{pK{a}_{i}}}{{10}^{pH}+{10}^{pK{a}_{i}}}-{\sum }_{j}{N}_{j}\frac{{10}^{pH}}{{10}^{pH}+{10}^{pK{a}_{j}}}$, where *N*_*i*_ are the number, and *pKa*_*i*_ the pKa values^63^, of the N-terminus and the basic side chains. The *j*-index refers to the C-terminus and side chains of Asp, Glu, Cys, and Tyr.

^‡^Aromatic and aliphatic residues.

^†^Apelin-55 used herein has a residual N-terminal Ser (underlined) from TEV protease-mediated tag removal.

The membrane catalysis theory, developed for peptide-receptor interactions by Sargent and Schwyzer^15^, states that peptide-receptor recognition and activation is preceded by binding between ligand and the plasma membrane. This initial peptide-membrane interaction “catalyses” the rate of ligand-receptor complex formation by confining the ligand to a 2-dimensional diffusion space, subsequently improving the diffusional encounter probability of the ligand-receptor pair. Furthermore, the membrane interaction increases local concentration of the ligand, and can induce structural change within the ligand for optimal receptor binding and activation. Thus, biological membranes have the potential to regulate ligand potency and efficacy, making the membrane a factor of particular relevance for ligand-receptor interactions. In support of this, numerous peptide hormones, including apelin and apela, have demonstrated binding to membrane-mimetics such as micelles and bicelles, leading to structural changes within the peptide^16–21^ (reviewed, e.g., by Langelaan and Rainey^22^).

We previously characterized apelin-membrane interactions, with a focus on the apelin-17 isoform^17^. Specifically, apelin-17 interactions were characterized with zwitterionic detergent micelles composed of dodecylphosphocholine (DPC); and anionic micelles composed of either sodium dodecyl sulfate (SDS) or 1-palmitoyl-2-hydroxy-*sn*-glycero-3-[phospho-*rac*-(1-glycerol)] (LPPG). A preferential interaction was demonstrated with anionic detergent micelles. It is important to note that DPC most closely mimics phosphatidylcholine, the most abundant phospholipid headgroup on the outer leaflet of eukaryotic plasma membranes^23^. SDS and LPPG, in contrast, are mimetic of either anionic lipids or glycolipids (with modifications conferring the negative charge) that may accumulate near the receptor^24–26^. In addition to demonstrating a preferential interaction with negatively charged micelles, our nuclear magnetic resonance (NMR) spectroscopy-based characterization demonstrated direct interaction between apelin-17 and anionic SDS micelle headgroups via the basic RPRL motif (Table 1), which adopted a type I β-turn conformation^17^. In addition, micelle binding led to highly converged structuring of the three C-terminal residues (MPF) in apelin-17, despite this region being completely solvent exposed. Structural characterization of the RPRL motif has since led to development of cyclic peptide analogues and competitive antagonists of the AR^27–31^.

We also recently characterized apela-32 and -11 in the presence of DPC, SDS, and LPPG micelles. Interestingly, apela demonstrated both isoform- and detergent headgroup-dependent changes in conformation and dynamics^21^. Notably, while both apela isoforms efficiently bound to SDS and LPPG micelles, they showed clear differences in their binding with DPC. Specifically, apela-32 readily bound to any of the three types of detergent micelles, while apela-11 demonstrated a significant preference for binding to anionic over zwitterionic micelles. Further elaboration came from the fact that zwitterionic headgroup binding was localized to the N-terminal half of apela-32, which adopted an α-helical conformation in response to binding. In combination, our findings on both apelin and apela indicate biological membrane as a potential regulator of the apelinergic system.

Interestingly, a number of studies have demonstrated length-dependent potency and efficacy for both apelin and apela. For example, apela-32 exhibited significantly higher potency than apela-11 for both receptor binding and signaling^4,5^, while apelin potency has generally been inversely correlated with increasing isoform size^9,32,33^. In terms of membrane catalysis, the differences in potency for apela can be justified by their differential interaction with membranes. Specifically, the favourable interaction with zwitterionic headgroup may lead to a greater level of apela-32 than -11 on the cell surface, which will consequently lead to higher probability of apela-32-AR binding/interaction and increased potency.

Although we compared micellar interactions of fluorophore-conjugated apelin-36 to apelin-17 and -12 conjugates, direct conclusions about apelin-36 could not be drawn because each conjugate was non-trivially distinct in its behaviour relative to either peptide alone or fluorophore alone^34^. To date, therefore, the longer apelin isoforms in their natural state remain uncharacterized in the presence of membrane-mimetics. As a consequence, it is unknown whether these isoforms differ in membrane interaction preferences. Furthermore, it is unknown whether longer apelin isoforms will interact similarly to shorter isoforms in a strictly headgroup-dependent manner or if the increased hydrophobic content of these peptides will allow for significant peptide interaction with the hydrophobic micelle core and, by extension, lipid tailgroups in a bilayer. Any, or all, of these potential discrepancies in interaction could give rise to the observed decrease in potency.

To test for the potential variation in membrane interaction between apelin isoforms, here we detail the biophysical characterization of the two longest isoforms, apelin-55 and -36, in the presence of DPC, SDS, and LPPG micelles. Apelin secondary structuring as a function of environment was characterized by far-UV circular dichroism (CD) spectropolarimetry alongside NMR spectroscopy to characterize both the relative prevalence of micellar interactions and changes in conformational equilibria and dynamics at the atomic-level.

## Results and Discussion

### Probing apelin-membrane interactions by CD spectropolarimetry

**Figure 1:**
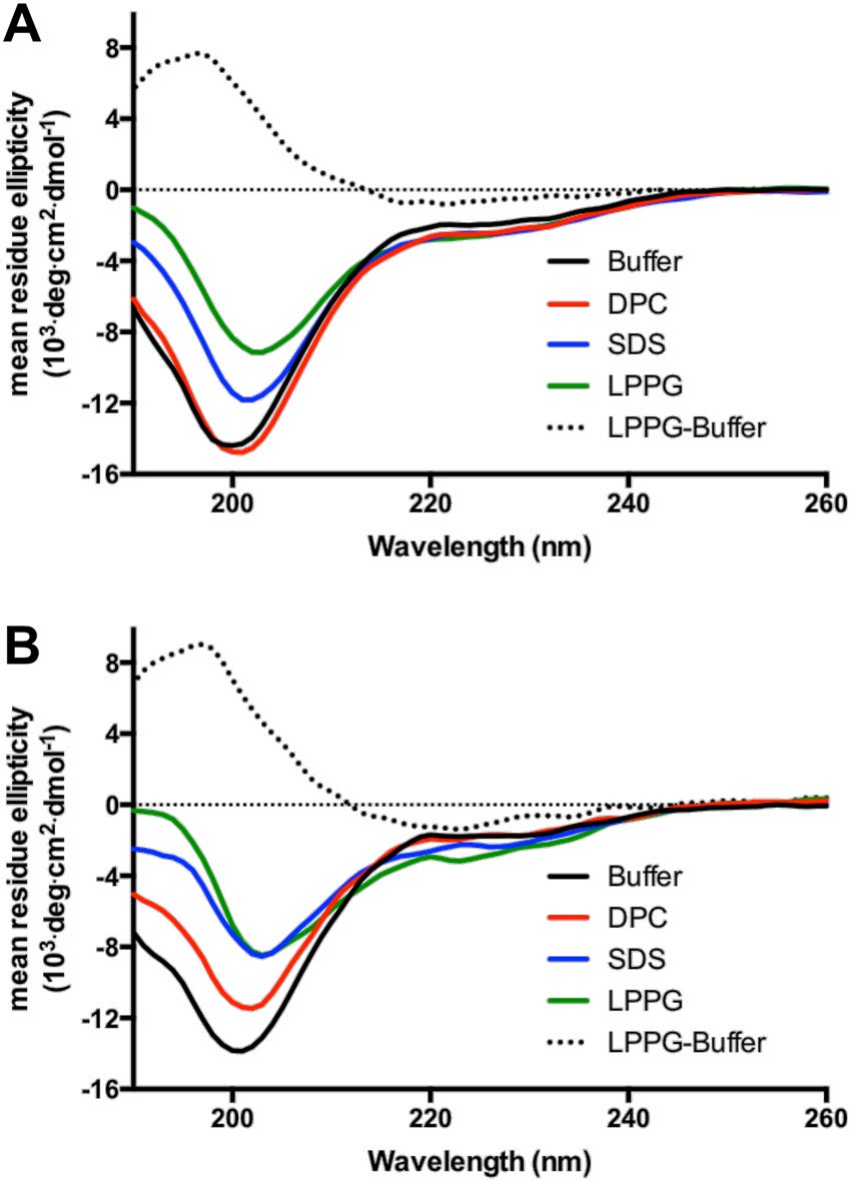
Apelin-micelle interaction characterized by far-UV CD spectropolarimetry for (**A**) apelin-55 and (**B**) apelin-36 at a 1:2 protein-to-micelle ratio for indicated micelle. LPPG-buffer is CD difference spectrum following subtraction of buffer spectrum from that in LPPG micelles.

Far-UV CD spectropolarimetry was used to test for perturbation to overall apelin peptide structuring in the presence of excess micelles (Fig. 1; ~1:2 protein:micelle stoichiometry, as was maintained throughout). Both apelin-55^9^ and -36^9,35,36^ have been demonstrated to have a random coil conformation in buffer, as indicated by a strong negative band at ~200 nm (e.g., Fig. 1). In the presence of zwitterionic DPC micelles, apelin-55 showed negligible spectral difference in comparison to buffer. However, apelin-36 showed an increase in ellipticity from 190–214 nm relative to its longer counterpart. This difference is clear upon spectral subtraction (Fig. 1 and Supplementary Fig. S1). Despite this minor difference, however, the minimal perturbation in the presence of DPC observed for both apelin-55 and -36 is highly similar to the behaviour of both apelin-17^17^ and apela-11^21^.

Contrasting with the behaviour in DPC, and echoing our findings with shorter apelin isoforms^17^, both apelin-55 and -36 exhibited a significant increase in ellipticity over 190–214 nm in the presence of anionic micelles. Difference spectra obtained in the presence of anionic micelles relative to buffer demonstrated a strong positive band at ~198 nm and a broad negative band at ~220 nm (dashed line, Fig. 1; Supplementary Fig. S1), which may be indicative of β-turn conformations^37,38^ or a non-canonical secondary structuring convolution. Consistent with this, spectral comparison of apelin-17 in anionic micelles relative to buffer showed similar positive band at ~198 nm and broad negative band at ~225 nm with unambiguous demonstration of β-turn induction by NMR spectroscopy^17^.

Notably, the structural perturbation observed for apelin-36 was more pronounced than that of apelin-55 in both anionic micelles (Fig. 1). Given units of mean residue ellipticity, where spectral magnitude is normalized by the number of peptide bond chromophores, this may result from a situation where the 36 C-terminal residues of apelin-55 undergo a disproportionally greater conformational change than the 19 N-terminal residues present only in apelin-55. Regardless of minor differences in the magnitude of ellipticity perturbation, both apelin-55 and -36 clearly exhibit preferable and more structurally perturbing interactions with anionic micelles relative to zwitterionic micelles, as was the case with apelin-17^17^. This contrasts with apela, where isoform-dependent behaviour was observed^21^.

### Comparison of apelin-micelle binding propensities

**Figure 2:**
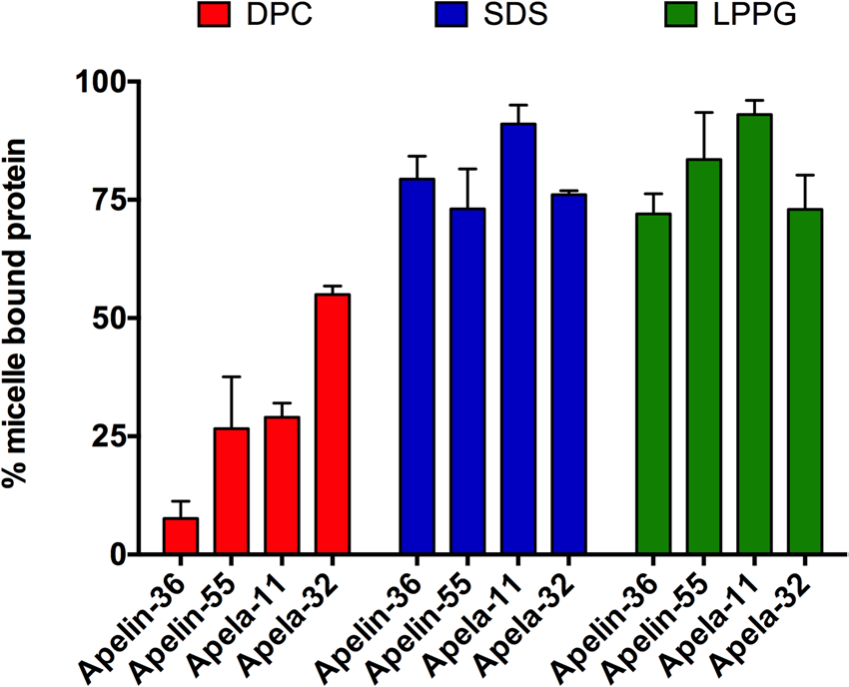
Percentage of micelle-bound apelin-36 and -55 population for the given condition. The percentages of micelle-bound apela-11 and -32 are re-plotted from Huang *et al*.^21^ to allow direct comparison to apelin isoforms.

Although apelin-micelle interactions are implied by CD spectroscopy, a more direct examination of binding is desirable. This was carried out using pulsed field gradient-based diffusion ordered spectroscopy (DOSY)^39^, which allows the determination of the translational diffusion coefficients. Under the assumption that a given apelin-micelle complex will diffuse more slowly than either the free ligand or micelle, observed diffusion coefficients (*D*_*ob*_) of apelin-55 and -36 in both the presence and absence of micelles, alongside those of each micelle in the absence of apelin, were determined and analysed (Table 2; experimental details in Supplementary Table S1). Notably, a decrease in *D*_*ob*_ was observed for both apelin-55 and -36 in all micelle conditions. The fraction of micelle-bound ligand (*f*_*b*_) was determined in each case using a two-state model (Fig. 2), as previously employed for apelin-17^17^ and apela-32 and -11^21^ micelle binding studies. In this model, apelin isoforms are assumed to sample two states under experimental conditions of an excess stoichiometric ratio of micelles relative to peptide: (i) free and (ii) micelle-bound. Consistent with the degree of perturbation evident from CD spectroscopy, the *D*_*ob*_ values were most severely reduced and the corresponding *f*_*b*_ were greatest in the presence of anionic micelles.

**Table 2:** Fraction of apelin-55 and -36 bound (*f*_*b*_) to the indicated micelles as determined by diffusion ordered spectroscopy (Values are presented as mean ± SEM).

Component	*η* (mPa·s)	*D*_*ob*_ (10^−10^ m^2^ s^−1^)^#^	*f*_b_^*^
No protein/micelle	0.712 ± 0.002	NA	NA
Free DPC micelle	0.746 ± 0.004	1.306 ± 0.001	NA
Free SDS micelle	0.753 ± 0.001	1.337 ± 0.004	NA
Free LPPG micelle	0.884 ± 0.003	0.817 ± 0.001	NA
Free apelin-55	0.715 ± 0.003	1.850 ± 0.006	NA
Apelin-55 with DPC micelle	0.738 ± 0.002	1.570 ± 0.009	0.266 ± 0.11
Apelin-55 with SDS micelle	0.738 ± 0.001	1.070 ± 0.004	0.731 ± 0.08
Apelin-55 with LPPG micelle	0.780 ± 0.002	0.796 ± 0.006	0.835 ± 0.10
Free apelin-36	0.711 ± 0.006	2.780 ± 0.006	NA
Apelin-36 with DPC micelle	0.756 ± 0.001	2.640 ± 0.003	0.076 ± 0.03
Apelin-36 with SDS micelle	0.739 ± 0.007	1.420 ± 0.000	0.794 ± 0.05
Apelin-36 with LPPG micelle	0.896 ± 0.002	1.300 ± 0.001	0.720 ± 0.04

^#^Experimental diffusion coefficients were determined using Eq. 1, and are reported as viscosity-corrected diffusion coefficients (*D*_*ob*_) using Eq. 4.

^*^Fraction of binding (*f*_*b*_) estimated using Eq. 3 under the assumption of two-state fast exchange.

DOSY-based D_ob_ determination relies upon quantification of diffusion-mediated signal decay. It is important to note, however, that signal decay may also occur through additional processes such as transverse relaxation. The rate of transverse relaxation (R_2_) increases with rotational correlation time (τ_C_)^40^. In the two-states of apelin being considered, the bound-state would thus be likely to experience a greater τ_C_, leading to a more rapid R_2_ for the bound-state vs. the free state. As this is unaccounted for in the model we have employed, the interaction between apelin isoforms and micelles is likely underestimated, with a greater degree of underestimation for the larger LPPG micelles vs. the similarly sized DPC and SDS micelles. Regardless of this underestimation, similar to shorter isoforms, the estimated *f*_*b*_ values demonstrate that both apelin-55 and -36 preferentially interact with anionic relative to zwitterionic micelles.

To test the potential for interaction with non-ionic detergent micelles, biophysical characterization of apelin-55 was also carried out in the presence Brij-35, the detergent employed in PCSK3 cleavage of apelin-55^10^. Apelin-55 was practically unperturbed in the presence of non-ionic Brij-35 micelles according to CD (Supplementary Fig. S2) and a correspondingly lower *f*_*b*_ than zwitterionic DPC micelles was observed (Supplementary Table S1). It is important to note that for larger micelles such as LPPG^41^ and Brij-35^42^, a decreased diffusion coefficient due to collision between ligands and micelles may become more apparent^43^ due to relative crowding. However, despite this potential error, the disproportionate decrease in *D*_*ob*_ of apelin-55 in response to LPPG relative to Brij-35 clearly indicates that apelin-membrane interaction is dependent on charge of detergent headgroups.

Comparison of binding behaviour of apela-32 and −11 to apelin-55 and -36 reveals striking differences. The most notable difference is between apelin-36 and apela-32 in DPC micelles. Specifically, although these isoforms are comparable in size and net charge (Table 1), they show large differences in their *f*_*b*_ value for zwitterionic micelles, while sharing relatively similar *f*_*b*_ values for anionic micelles. In addition, the variability in *f*_b_ for zwitterionic micelles indicates that the hydrophobic effect and corresponding partitioning into the micelle interior is likely a minimal mediator of apelin-micelle interaction, as the *f*_*b*_ values observed in all micelle types for apelin-55 with the largest hydrophobic content (Table 1) and apela-11 with the lowest hydrophobic content were very similar (Fig. 2). Interestingly, for both apelin and apela, increasing isoform size resulted in an increase in *f*_*b*_. Collectively, these results indicate that electrostatic interactions are likely the main contributor to apelin-micelle interaction with favourable interaction occurring between positively charged residues of apelin isoforms and negatively charged micelle headgroups.

### Chemical shift assignment of apelin-55 in micellar environments

**Figure 3:**
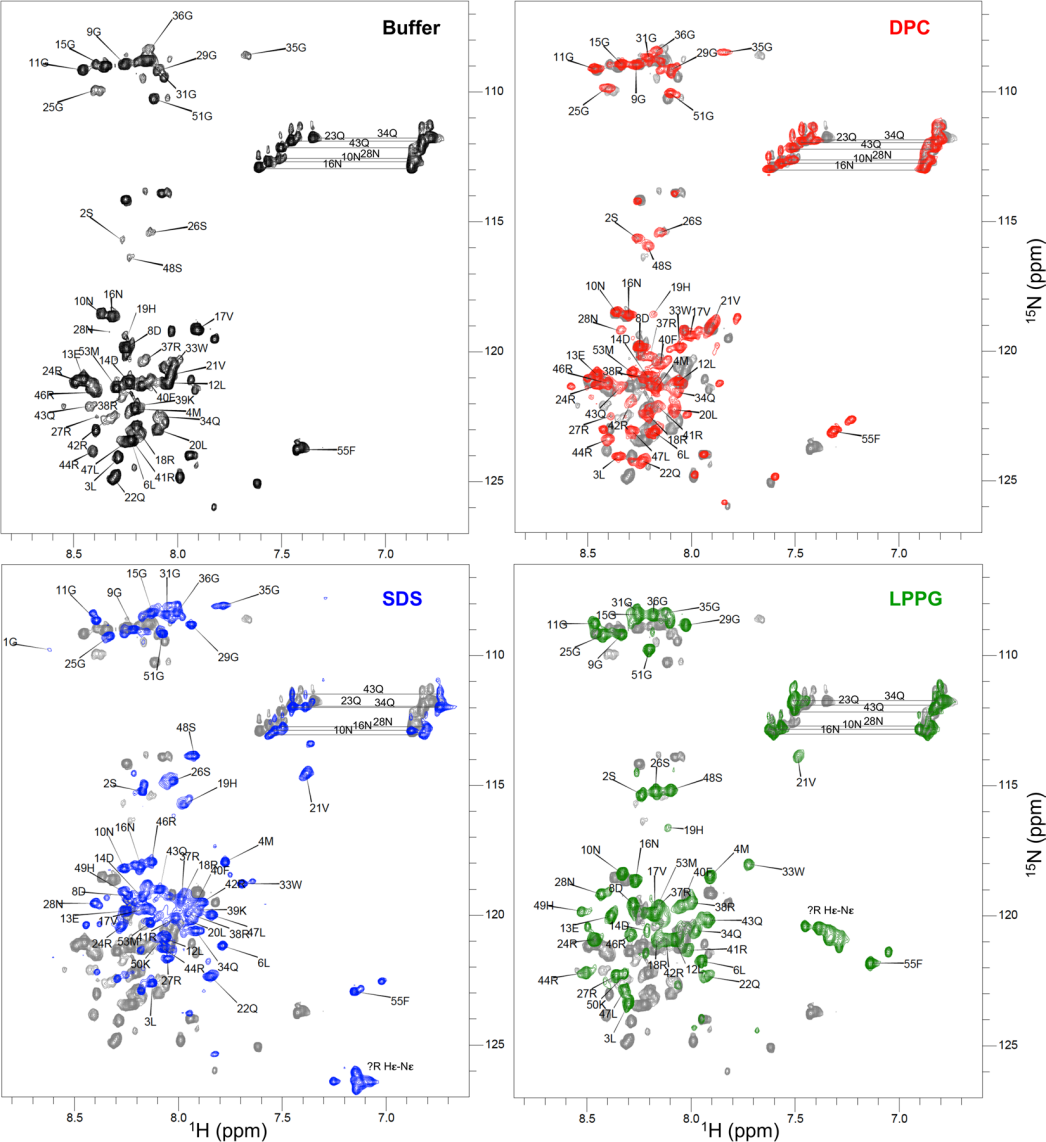
^1^H-^15^N HSQC spectra of apelin-55 in indicated conditions with cross-peaks annotated by number in the sequence and single-letter amino acid code for primary sequentially-assigned chain (minor peaks are annotated in Supporting Information). The data for apelin-55 in buffer were previously reported^9^ and are plotted here for direct comparison; spectra of apelin-55 in micellar conditions (coloured) are overlaid on the spectrum in buffer (grey).

The chemical shifts of nearly all (Table 3) apelin-55 backbone (N, H_N_, C_α_, and C′) and C_β_ nuclei alongside Asn N_δ_ and H_δ_ and Gln H_ε_ and N_ε_ were assigned in DPC, SDS, and LPPG micelle conditions using standard triple-resonance NMR experiments (chemical shifts detailed Supplementary Tables S2–4; comparative backbone walk segment shown in Supplementary Fig. S3). For reference, H_N_-N resonance assignments are annotated on the corresponding ^1^H-^15^N heteronuclear single quantum coherence (HSQC) spectra (Fig. 3, with additional minor peak assignments denoted in Supplementary Fig. S4). Despite the spectral perturbations observed by CD spectroscopy, chemical shift analysis of apelin-55 in each of the three micelle conditions showed only minor differences to expected random coil chemical shifts. Furthermore, analysis of the assigned chemical shifts by the chemical shift index (CSI^44^) and the Bayesian algorithm Dihedral Angles from Global Likelihood Estimates (DANGLE^45^) did not indicate any segments of extended secondary structuring in any of the experimental conditions (Supplementary Fig. S5).

**Table 3:** Degree of completion of chemical shift assignment relative to expected total number assignable for a given atom for apelin-55 in each indicated condition.

Atom type	Buffer^§^	DPC	SDS	LPPG
H_N_*	44/47 (94%)	43/47 (91%)	47/47 (100%)	44/47 (94%)
N^‡^	44/47 (94%)	43/47 (91%)	47/47 (100%)	44/47 (94%)
C′	54/56 (96%)	54/56 (96%)	56/56 (100%)	53/56 (95%)
C_α_	54/56 (96%)	54/56 (96%)	55/56 (98%)	55/56 (98%)
C_β_	44/46 (95%)	40/46 (87%)	42/46 (91%)	34/46 (74%)

^§^Apelin-55 in buffer at 37 °C is based on chemical shifts reported in Shin *et al*.^9^.

^*^N-terminal H_N_ is excluded from counts.

^‡^Proline residues and N-terminal N are excluded from counts.

In all three micelle conditions, 0S (the additional residue at the N-terminus of our apelin-55 following TEV protease cleavage) was unassignable, likely due to fast exchange of the H_N_ with solvent^46^. 1G was only assignable in the presence of SDS, potentially due to a micelle headgroup-dependent protection from exchange with solvent. In the presence of SDS and LPPG, but not DPC, Arg side chain H_ε_-N_ε_ resonances were also observed; these could not be unambiguously assigned.

Beyond these N-terminal residues, three other regions exhibited headgroup-dependent variations in behaviour. 49H and 50K were not assignable in DPC, as was also the case in our recent characterization of apelin-55 in buffer^9^. The lack of assignable resonances for these residues was attributed to increased conformational flexibility in this region of apelin, thus falling within NMR timescale of intermediate exchange, as observed in our characterization of apelin-17^36^. This finding is, thus, indicative of similar dynamics over this segment being experienced by apelin-55 in DPC micelles and in buffer, corresponding to a low degree of micellar interaction. 39K was, conversely, not assignable in either DPC and LPPG micelles, although this residue was assignable in buffer^9^ and in SDS micelles. The lack of assignable resonances for 39K for these micelle conditions is likely the result of a localized change in dynamics into the intermediate exchange regime on the NMR timescale in response to DPC and LPPG micelle binding. Lastly, 20L could not be assigned in LPPG, potentially due to differences in local dynamics in response to this micelle.

In our previous characterization of apelin-55 in buffer, we observed additional spin-system assignments for a number of residues, indicating sampling of multiple conformational states in the slow exchange regime^9^. Interestingly, the number of potential assignments per residue changed as a function of micelle type (Supplementary Figs S4–S5), suggesting alterations in both the number and equilibria of the conformations being sampled in the slow exchange NMR time-scale. Similar to the buffer state at 37 °C^9^, there were no stretches of additional sequential assignments possible (>2 residues) for all three micelle conditions consistent with additional major conformations.

### Membrane headgroup-dependent conformational and dynamic changes for apelin

**Figure 4:**
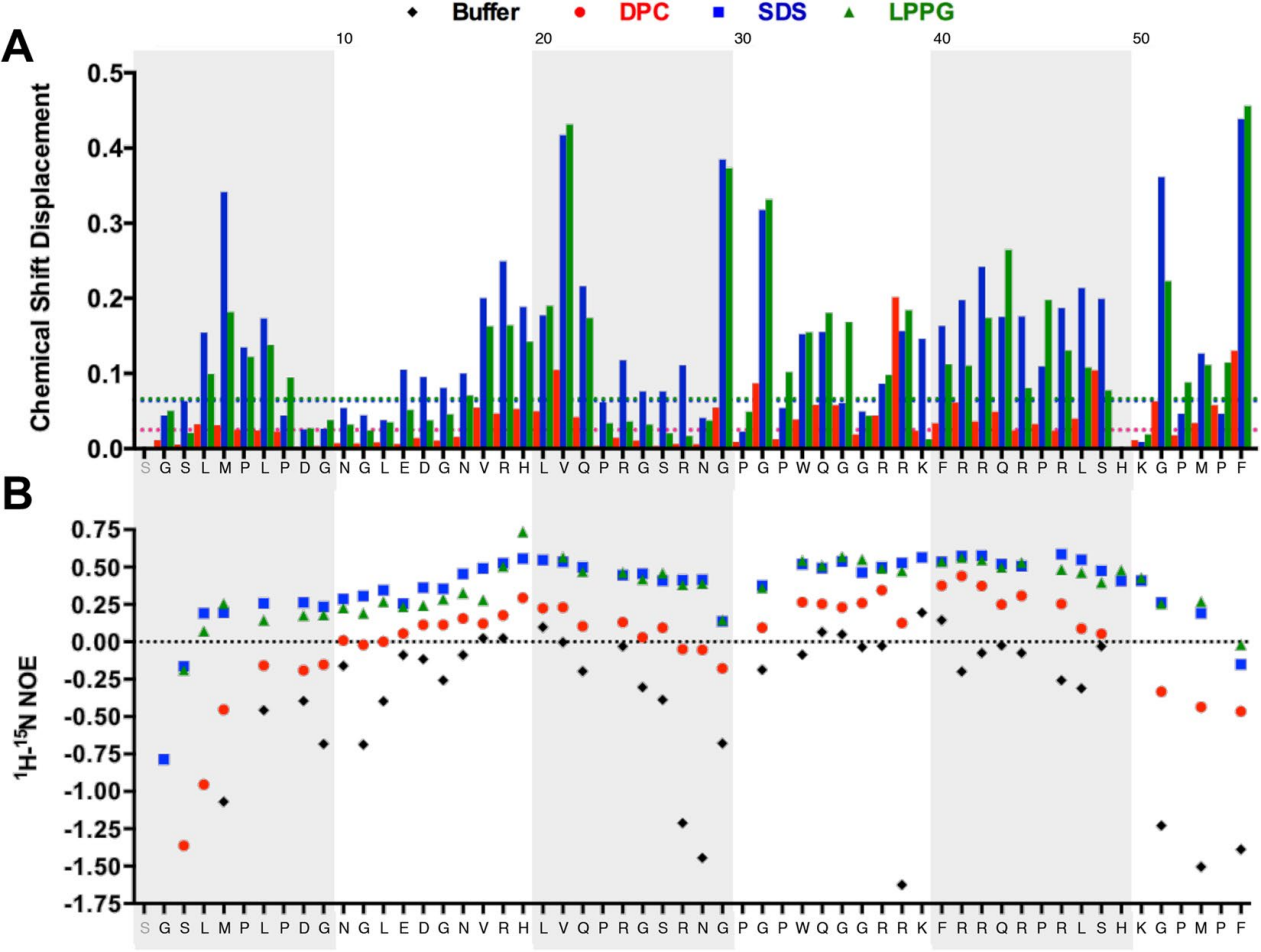
Micelle headgroup-dependent conformational and dynamic changes in apelin-55. (**A**) Chemical shift displacement (H_N_, N, C_α_, C′) and (**B**) ^1^H-^15^N heteronuclear NOE enhancement factor in presence of indicated micelle relative to previously assigned buffer chemical shifts^9^. Dashed lines represent corrected standard deviation (σ_0_) of chemical shift displacement values. To allow direct comparison, the ^1^H-^15^N heteronuclear NOE enhancement factors previously reported in buffer^9^ are shown.

Although there were only minor perturbations relative to expected random coil chemical shifts and no indication of extended secondary structural elements in each micellar condition (Supplementary Fig. S6), the Euclidian combined chemical shift displacement (CSD) for N, H_N_, C_α_, and C′ for apelin-55 varied as a function of micelle type (Fig. 4A), with the lowest magnitude observed for DPC micelles and higher magnitudes observed for SDS and LPPG micelles. This is consistent with a fast exchange between bound and free states. Specifically, fast exchange would result in the observation of a single resonance frequency for a given nucleus with chemical shift resulting from the weighted average of proportion of free and bound states, while slow exchange would be reflected in two resonance frequencies with chemical shifts corresponding to the free and bound states with the intensity of each modulated by the proportion of the state^47^. Hence, the observation of a correlation between the magnitude of CSD and *f*_b_ of apelin-55, with ~27% bound in DPC relative to 73% and 84% bound in SDS and LPPG, respectively, is indicative of a fast exchange process rather than a slow exchange process.

Restriction in ps-ns timescale dynamics was also clearly observed in each of the micellar conditions along the apelin-55 backbone, as evidenced by increases in the ^1^H-^15^N heteronuclear nuclear Overhauser effect (NOE) enhancement factors relative to buffer. Both anionic micelles exhibited similar increases in heteronuclear NOE enhancement factor that were more pronounced than DPC, with DPC exhibiting enhancement factors falling between those with anionic micelles and those in buffer (Fig. 4B).

These CSD and heteronuclear NOE enhancement data are both, therefore, consistent with the CD spectroscopy and DOSY data indicating a greater degree of apelin-55 structural perturbation by and interaction with anionic micelles than by/with DPC. It should be noted that although both anionic micelles caused significant changes of similar magnitude in both CSD (Fig. 4A) and heteronuclear NOE enhancement (Fig. 4B) for apelin-55, distinct ^1^H-^15^N HSQC cross-peak patterns were observed (Fig. 3). This suggests that the conformations and/or local chemical environments resulting from micelle interaction are dependent on the detergent headgroup properties.

Comparison of apelin-55 chemical shifts in all three micelles relative to buffer suggest a number of regions are likely involved in micellar interaction, with the magnitude of CSD approximately scaling with *f*_*b*_ in each of these regions. Specifically, the 3L-6L, 17V-22Q, 29G-34Q, and 37R-48S regions all demonstrate comparatively large CSD values relative to other residues of apelin-55 (Fig. 4A). All of these regions contain aromatic and/or basic residue(s), providing the potential for micellar interaction through the hydrophobic tailgroup (for aromatics) and/or polar/charged detergent headgroups (aromatic or basic). Notably, the apelin-55 N-terminal regions which showed interactions may explain the increased *f*_b_ for apelin-55 in DPC and LPPG relative to apelin-36. As apelin-55 encompasses more micelle-interacting segments than its shorter counterpart, the bound population may increase accordingly.

**Figure 5:**
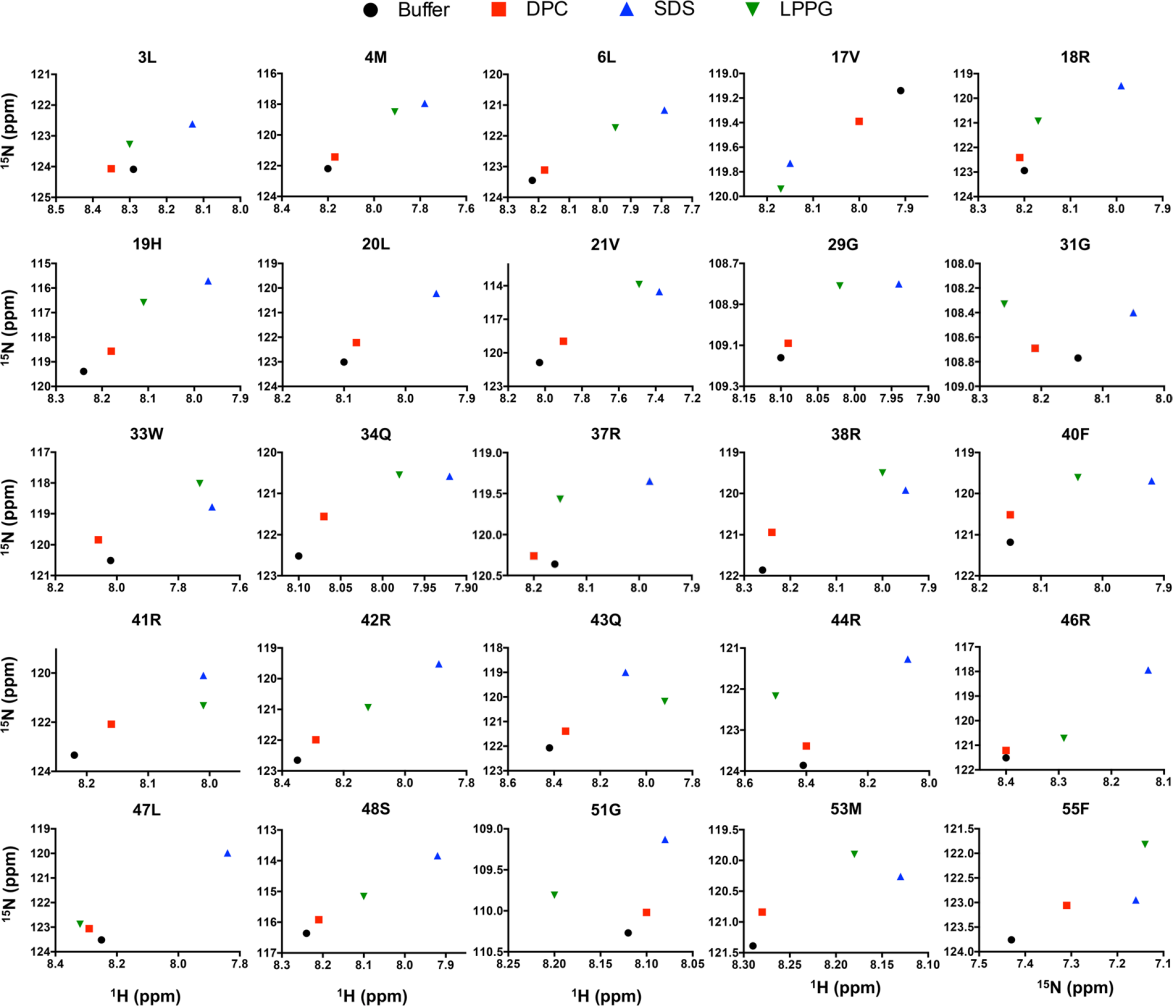
Micelle-dependent modulation of assigned H_N_ and N resonances for indicated residues.

Given the relatively overlapped nature of the ^1^H-^15^N HSQC spectra (Fig. 3), ^1^H and ^15^N resonance assignments for residues in each of the perturbed regions were plotted individually as a function of micelle condition (Fig. 5). Notably, these regions appear to exhibit similar patterns of CSD but with differing magnitudes. The majority of these perturbed residues exhibit behaviour consistent with a two-state fast exchange^47^, as introduced above, where the observed chemical shift is a weighted function of the relative proportion of peptide bound in a given micellar condition (Fig. 2). This is not uniformly observed. Such disparate behaviour would be fully expected if a given amide bond is in closer proximity to the detergent headgroup in the bound state or in a dissimilar interfacial or hydrophobic core regions of the micelle. This could, in turn, be influenced by an altered conformational equilibrium in response to size and/or fluidity of the micelle or by a change in partitioning coefficient of hydrophobic segments into the micelle interior with a change in the hydrophobic core. Given the disparate dipole moments, charge distributions, steric bulk, and tailgroups of the detergents employed (detailed, e.g., in Patterson *et al*.^34^), such residues would experience different and additional chemical shift perturbations. These data are, thus, consistent with a situation where there is a similar bound-state conformation (or, likely more accurately, equilibrated set of conformations) undergoing fast exchange with the free state regardless of the micelle type. It should be noted that this interpretation was only feasible through sequential chemical shift assignment in each condition, allowing comprehensive analysis of H_N_ and N chemical shift modulation at each residue as a function of both *f*_*b*_ and headgroup.

### Backbone-level independence of apelin-36

In examination of apelin-55 interactions with DPC, SDS, and LPPG micelles, segregation of the N-terminal and C-terminal regions is apparent, where the average CSD observed for the 36 C-terminal residues (common to apelin-36) is greater than that of the 19 N-terminal residues exclusive to apelin-55. The heteronuclear NOE enhancement is also indicative of a lesser degree of ps-ns timescale dynamics in the C-terminal region relative to the N-terminal region. This corresponds well to the observed greater perturbation in mean residue ellipticity for apelin-36 vs. apelin-55, with more conformational change being observed in the 36 C-terminal residues shared between isoforms than in the 19 N-terminal residues present only in apelin-55.

**Figure 6:**
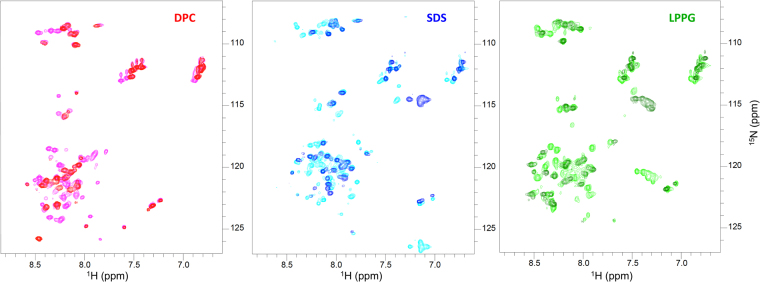
^1^H-^15^N HSQC spectra of apelin-36 (red, blue, and green) overlaid on those of apelin-55 (magenta, cyan, and lime) in the indicated micellar condition.

To test the hypothesis that the apelin-36 segment behaves similarly whether in the context of apelin-55 or in isolation, apelin-36 was also characterized by NMR spectroscopy. ^1^H-^15^N HSQC spectra exhibited nearly identical perturbation patterns between the two isoforms in all three micelles (Fig. 6). The observation of virtually unchanged ^1^H-^15^N HSQC spectra also allowed for direct inference of cross-peak assignments for apelin-36 based on the longer 55-residue counterpart (Supplementary Figs S7–9). The only exception to this was the four N-terminal residues, for which no assignable spin-systems were observed, likely as a result of increased exchange with solvent and, potentially, intermediate exchange^47^ on the NMR timescale.

Direct quantitative comparison of H_N_ and N chemical shifts between apelin-55 and apelin-36 demonstrates that the vast majority of residues exhibit a CSD of less than 0.04 ppm (Supplementary Fig. S10; chemical shifts for apelin-36 presented in Supplementary Table S6). This echoes the fact that all of the bioactive apelin isoforms (i.e., apelin-13, -17, -36, and -55) maintain an identical conformational equilibrium for the shared region in buffer^9^. The minimal CSD values observed are consistent not only with the adoption of a highly similar conformation by both isolated apelin-36 and the corresponding segment in apelin-55 in response to each micelle but, more strikingly, with highly comparable fast exchange regime sampling of bound and free states. Notably, despite being the least perturbed overall relative to the buffer state, apelin-36 in DPC exhibits the highest CSD compared to apelin-55 of the three micellar conditions. Given that *f*_*b*_ is most disparate between apelin-36 and -55 in DPC (Fig. 2), this modestly larger CSD is logical as this would lead to a perturbation to the free vs. bound state equilibrium underlying the observed average chemical shift.

The highly similar conformation sampling behaviour of both apelin isoforms in all three micelle conditions is in striking contrast to apela isoforms. Specifically, apela-11 showed chemical shift differences in comparison to apela-32, impeding H_N_-N resonance assignment through chemical shift inference^21^. This implied that apela-32 and -11 have differing conformations due to interaction between the N-terminal region of apela-32 and the C-terminal region corresponding to apela-11. In addition, this difference in behaviour was further exemplified in the presence of zwitterionic DPC micelles. The α-helical segment induced in the N-terminal region of apela-32 (vide supra), a region absent in apela-11, likely serves to stabilize its interaction with DPC. Converse to this, the high degree of chemical shift similarity between apelin-55 and -36 in buffer^9^ and in all three micellar environments suggests that there are no apparent stabilizing interactions between N- and C-terminal domains in the longer apelin isoforms, even in the presence of micelles.

### Implications of membrane catalysis in the apelinergic system

Membrane catalysis may explain the increased potency for apela-32 in comparison to apela-11^4^ and apelin-13^5^, given the relatively high propensity for apela-32 interaction with DPC micelles (Fig. 2, as detailed in Huang *et al*.^21^) that serve as a mimic for the most prevalent lipid in typical eukaryotic cells. The isoform-dependent differences in potency between apelin isoforms cannot be similarly explained. Although apelin-17^17^, -36, and −55 all preferentially interact with anionic over zwitterionic micelles, the data presented herein show that peptide behaviour in the bound state is similar regardless of headgroup properties, with the NMR observables being scaled by the proportion of bound vs. free peptide.

Notably, the relatively large CSD observed in the 37R-48S region is consistent with our previous characterization of apelin-17 (vide supra) suggesting that longer apelin isoform may also bind to anionic detergent headgroup through the RPRL motif. If so, the three C-terminal residues (MPF) are likely structurally converged in apelin-55 upon anionic micelle binding. In support of this, a significant loss in dynamics by heteronuclear NOE enhancement was observed for 53 M and 55 F (Fig. 4B), while clearly exhibiting behaviour consistent with fast exchange between free and micelle-bound state proportional to *f*_b_ (Fig. 5). These findings collectively imply that the functionally critical C-terminal RPRL and MPF motifs adopt a similarly converged structuring as apelin-17, translating to a similar mode of membrane-catalysed receptor activation.

Clear observation of fast exchange between the free and bound states of apelin for all micelles indicates that binding and unbinding rates are on the order of 10^5^ s^−1^ or faster^48^, with a similar bound-state conformation being sampled by the proportion of ligand molecules on the membrane. A lower proportion of peptide bound to zwitterionic headgroups at any given time would allow for a less hindered diffusional search for the receptor, while also maintaining a high local concentration proximal to the membrane. Hence, one could envision a situation where the ligand rapidly traverses the predominantly zwitterionic extracellular face of the membrane, frequently sampling the bound state conformation, with an increased propensity for membrane binding and association in areas of elevated anionic character. Given the shared C-terminal region of all apelin isoforms and membrane interaction behaviour, highly similar membrane-catalysed receptor recognition and binding would be facilitated regardless of either the length of the isoform or the local lipid environment. Correspondingly, apelin isoform-dependent differences in potency may not be directly regulated by membrane interaction. Instead, these seem likely to be mediated by changes in the receptor conformational equilibrium in response to ligand, following membrane-catalysed binding, potentially through interaction with recently identified^49^ anionic grooves on the AR surface that are unoccupied by an apelin-17 analogue. Thus, future experiments should focus on delineating the common structural and dynamic features of apelin and apela isoforms that are involved in the membrane-catalysed ligand-receptor recognition step versus features that are involved in activation and downstream regulation.

The membrane catalysis theory does not explicitly consider the roles of interactions with molecules beyond the membrane surface. This is an important omission, as these molecules may act as binding partners, hypothetically inhibiting interaction between apelin and the membrane surface. Steric interference may also decrease peptide-membrane interaction. Membrane surfaces are also composed of diverse types of lipids, some with complex post-translational modifications, and are highly dynamic^50^. Despite the simplification arising from use of membrane-mimetic micelles, the distinct apelin-micelle interactions observed clearly imply the potential for membrane catalysis to regulate apelin activity. Furthermore, this sets the foundation for future experiments exploring apelin and apela membrane interactions in increasingly physiological conditions, such as bicelles and liposomes, including characterization of the effects of species (i.e., other extracellular molecules) that may compete or interfere with the peptide-membrane interaction.

## Materials and Methods

### Materials

Ampicillin, SDS, acetonitrile (high performance liquid chromatography (HPLC) grade), and reagents to make lysogeny broth (LB) medium were purchased from Fisher Scientific (Ottawa, ON). DPC and LPPG were purchased from Anatrace (Maumee, OH) and Avanti Polar lipids (Alabaster, AL), respectively. ^15^NH_4_Cl, ^13^C_6_ -d-glucose, SDS-d_25_, and DPC-d_38_ were purchased from Cambridge Isotope Laboratories (Tewksbury, MA). Deuterium oxide (D_2_O; 99.8 atom % D) and D_2_O containing 1% (w/w) sodium 2,2-dimethyl-2-silapentane-5-sulfonate (DSS) were obtained from C/D/N Isotopes (Pointe-Claire, QC). All other reagents were purchased from Sigma-Aldrich Canada (Oakville, ON).

### Apelin-55 and -36 preparation

Human apelin-55 was expressed with an N-terminal His_6_-tag followed by a TEV protease cleavage site in *Escherichia coli* C41 (DE3). The His_6_ + TEV cleavage site-tagged apelin-55 was purified initially using Ni-NTA affinity, with the tag removed using TEV protease (prepared in-house), as detailed previously^10^. Tag-free apelin-55, with an N-terminal Ser remaining from TEV cleavage (total 56 residues, with the additional Ser referred to as residue 0 herein) was further purified by cation exchange chromatography followed by reverse-phase HPLC (RP-HPLC), as outlined previously^10^. Human apelin-36 was expressed with an N-terminal His_6_-SUMO fusion tag in *E. coli* BL21 (DE3). Ni-NTA affinity was employed to purify the fusion protein, with subsequent His_6_-SUMO tag removal using SUMO protease (prepared in-house). Finally, apelin-36 was purified by RP-HPLC, as detailed previously^9^. Aliquots of purified peptides at a given concentration (c) were quantified prior to lyophilisation using the Beer-Lambert law (c = A•ε^−1^•l^−1^, where A is absorbance at a given wavelength, ε is molar absorptivity at that wavelength, and l is pathlength). For apelin-36 and -55, absorbance at 280 nm was employed (ε of 5,500 M^−1^cm^−1^ estimated following validated protocols^51^).

### CD spectropolarimetry

Far-UV CD spectra of apela-55 and -36 were recorded at 37 °C using a J-810 spectropolarimeter (Jasco, Easton, MD) with 0.1 mm quartz cuvettes (Hellma, Vaughan, ON). Lyophilized peptide samples were diluted in an appropriate volume of CD buffer (1 mM NaN_3_, 1 mM CaCl_2_, 25 mM Na_2_HPO_4_/NaH_2_PO_4_ buffer, pH 6.00 ± 0.05) to obtain a final concentration of 0.2 mM. For micelle interaction studies, lyophilized aliquots of apelin-55 or apelin-36 were resuspended in CD buffer containing 32 mM SDS, 38 mM DPC, 76 mM LPPG, or 16 mM Brij-35 to achieve a 1:2 protein:micelle ratio based on aggregation numbers and critical micelle concentrations previously reported^41,52,53^. CD spectra were acquired in triplicate from 260 nm to 180 nm at 100 nm/min with a data pitch of 0.1 nm, with each experiment performed in duplicate using independently prepared samples. Ellipticity values were averaged, blank-subtracted, converted to mean residue ellipticity, and subjected to a 3 nm sliding window average.

### DOSY and Peptide-Micelle Binding Analysis

For DOSY, uniformly ^15^N-enriched apelin-55 and -36 were prepared at 0.2 mM in NMR buffer (1 mM DSS, 1 mM NaN_3_, 25 mM Na_2_HPO_4_/NaH_2_PO_4_ buffer, pH 6.00 ± 0.05, 90%/10% H_2_O/D_2_O). For micelle interaction, lyophilized aliquots were dissolved in NMR buffer containing 32 mM SDS (SDS-d_25_ for apelin-36), 38 mM DPC (DPC-d_38_ for apelin-36), 76 mM LPPG, or 16 mM Brij-35 similar to CD spectroscopy conditions to achieve an ~1:2 protein:micelle ratio. Identically prepared micelle samples without apelin incorporation were also analysed. All experiments were conducted at 37 °C using an Avance 500 MHz spectrometer equipped with a room temperature 5 mm broadband fluorine observe (BBFO) SmartProbe with a z-axis gradient (Bruker Canada; NMR^3^ facility at Dalhousie University, Halifax, NS). Supplementary Table S7 details experimental parameters. All samples for DOSY analysis were prepared in symmetrical susceptibility-matched NMR microtubes (Shigemi, Allison Park, PA). Stimulated echo ^1^H and ^31^P DOSY experiments with bipolar gradients and longitudinal eddy-current delay^51^ were employed to collect pseudo two-dimensional data (F1: one-dimensional ^1^H/^31^P NMR spectra; F2: corresponding diffusion coefficient) with individually optimized gradient length (𝛿) and diffusion time (Δ) (Supplementary Table S1). A DOSY-HSQC experiment was also employed to allow for straightforward and unambiguous characterization of ^15^N-enriched protein diffusion (led1dhsqc2d.jkr; Bruker TopSpin pulse program available upon request). Specifically, a 1D diffusion-editing experimental block using stimulated echo and longitudinal eddy-current delay with bipolar gradient pulses (Bruker pulse program ledbpgp2s1d^51^) was inserted in place of the initial 90° ^1^H pulse of a sensitivity enhanced ^1^H-^15^N HSQC acquired with water flip back pulses and ^15^N decoupling during acquisition (Bruker pulse program hsqcetfpgpsi2^54–57^. To perform DOSY, a series of 1D ^1^H-^15^N HSQC experiments were carried out with increasing diffusion measurement gradient amplitude (parameters detailed in Supplementary Table S1). Translational diffusion coefficients for each diffusing species in a given DOSY experiment were determined using the diffusion module of Dynamics Centre (Bruker) based on the relationship:

1 $$I={I}_{0}\,{e}^{-D{{\rm{g}}}^{2}{\gamma }^{2}{\delta }^{2}({\rm{\Delta }}-\frac{\delta }{3})}$$

where *I* is the observed intensity at a given value of gradient strength and *I*_0_ is the intensity at 2% gradient strength. D is the diffusion coefficient of the selected NMR signals, g is the gradient strength, and γ is the gyromagnetic ratio of the nucleus in question (^1^H or ^31^P). Lastly, δ and Δ are the gradient length and diffusion time, respectively. Diffusion coefficients were obtained by integrating two to three separate regions of the NMR spectrum for DOSY or DOSY-HSQC experiments, with two replicates averaged for the higher sensitivity ^1^H- and ^31^P-DOSY experiments.

Apelin-micelle binding was quantified using a two-state model, as introduced previously^21^. Briefly, assuming an excess of micelles (due to ~2:1 stoichiometry of micelle to peptide), peptide-micelle binding was considered as an equilibrium between free and bound peptide states in fast exchange, allowing the diffusion coefficient of the bound complex (*D*_*b*_) to be estimated from those of the free peptide (*D*_*p*_) and the free micelle (*D*_*m*_):

2 $$\frac{1}{{D}_{b}}=\frac{1}{{D}_{p}}+\frac{1}{{D}_{m}}$$

The ensemble-averaged diffusion coefficient observed for the peptide population (*D*_*ob*_) is then given by the sum of the fraction of peptide in the micelle-bound state (*f*_*b*_) and free states (1 − *f*_*b*_) with respect to the appropriate diffusion coefficient^58^:

3 $${D}_{ob}={f}_{b}{D}_{b}+(1-{f}_{b}){D}_{p}$$

For *D*_*ob*_, *D*_*p*_, and *D*_*m*_, the diffusion coefficients obtained over all spectral regions and/or replicates were averaged and normalized for viscosity variation using the equation:

4 $${D}_{ob,p,m}={D}_{sample}\cdot \frac{{\eta }_{sample}}{{\eta }_{buffer}}$$

where *η*_*sample*_ and *η*_*buffer*_ are the viscosities of a given sample and of buffer alone, respectively. Viscosities were determined at 37 °C using a temperature controlled *micro*Visc HVROC-L microviscometer (RheoSense, San Ramon, CA).

### Triple-resonance NMR spectroscopy and isoform comparison analysis

For triple-resonance NMR experiments, uniformly ^13^C- and ^15^N-enriched apelin-55 samples were prepared at 0.4 mM in NMR buffer with 64 mM SDS-*d*_25_, 76 mM DPC-*d*_38_, or 152 mM LPPG. Samples were prepared in symmetrical susceptibility-matched NMR microtubes shaped for CryoProbe usage (Bruker, Fällanden, Switzerland). Sensitivity enhanced ^1^H-^15^N HSQC and ^1^H-^15^N HSQC experiments acquired in an interleaved fashion with or without ^1^H saturation during the recycle delay (5 s) to determine the intensity of the ^1^H-^15^N heteronuclear NOE enhancement for a given HSQC cross-peak were acquired for each condition with ^13^C decoupling applied. Triple-resonance experiments for backbone assignment (HNCA, HNCoCA, HNCO, HNCaCO, and CBCANH or HNCACB) were also carried out for each micellar condition, allowing assignment of chemical shifts for the C′, C_α_, C_β_, H_N_, and N nuclei through the main chain-directed approach^40^. These data were acquired using an Avance III 700 MHz NMR spectrometer equipped with a 5 mm triple resonance inverse (TCI) cryoprobe with a z-axis gradient (Bruker Canada; Biomolecular Magnetic Resonance Facility, National Research Council, Halifax, NS). All experiments employed standard pulse programs from the TopSpin 2.1 library (Bruker Canada, Milton, ON; Supplementary Table S7 details experimental parameters). For isoform comparison, uniformly ^15^N-enriched apelin-36 were prepared in the same experimental conditions as DOSY experiments (vide supra) and ^1^H-^15^N HSQC spectra were acquired using an Avance 500 MHz spectrometer equipped with a BBFO SmartProbe (Bruker Canada). Supplementary Table S7 details experimental parameters.

Spectra were processed using TopSpin 3.1 and NMRPipe^59^. ^1^H frequencies were referenced to DSS (0 ppm) and ^15^N and ^13^C frequencies were referenced indirectly to^1^H^60^. Sequential residue assignments, DANGLE predictions^45^, CSI^44^, secondary chemical shifts (Δδ), and heteronuclear NOE intensity ratio calculations (intensity ratio of a cross-peak in the saturated relative to unsaturated interleaved experiments) were performed using CcpNmr Analysis 2.4.2^61^. The identified H and H_N_ chemical shifts for apelin-55 were used to assign ^1^H-^15^N HSQC spectra of apelin-36 by inference in the corresponding micelle conditions. CSD values were calculated as detailed previously^62^. Specifically, CSD values were determined using N, H_N_, C_α_, and C′ chemical shifts of apelin-55 in micelle conditions relative to our previously reported apelin-55 chemical shifts in buffer^9^ while the CSD based upon N and H_N_ chemical shifts of apelin-36 were compared to apelin-55 for each micelle condition. Corrected standard deviation (σ_0_) of CSD was determined using a modification of the approach detailed by Schumann *et al*.^62^. Specifically, the standard deviation (σ) of the shift changes were calculated and any residues with shift changes greater than 3σ were excluded. The remaining shift changes were used to calculate the σ_0_.

## Electronic supplementary material

Supplementary Information

## References

[1] Chapman NA, Dupre DJ, Rainey JK (2014). The apelin receptor: physiology, pathology, cell signalling, and ligand modulation of a peptide-activated class A GPCR. Biochem Cell Biol.

[2] Wang Z (2015). Elabela-apelin receptor signaling pathway is functional in mammalian systems. Sci Rep.

[3] Perjes A (2016). Characterization of apela, a novel endogenous ligand of apelin receptor, in the adult heart. Basic Res Cardiol.

[4] Murza A (2016). Discovery and structure-activity relationship of a bioactive fragment of ELABELA that modulates vascular and cardiac functions. J Med Chem.

[5] Deng C, Chen H, Yang N, Feng Y, Hsueh AJ (2015). Apela regulates fluid homeostasis by binding to the APJ receptor to activate G_i_ signaling. J Biol Chem.

[6] Chng SC, Ho L, Tian J, Reversade B (2013). ELABELA: a hormone essential for heart development signals via the apelin receptor. Dev Cell.

[7] Pauli A (2014). Toddler: an embryonic signal that promotes cell movement via apelin receptors. Science.

[8] Mesmin C, Fenaille F, Becher F, Tabet JC, Ezan E (2011). Identification and characterization of apelin peptides in bovine colostrum and milk by liquid chromatography-mass spectrometry. J Proteome Res.

[9] Shin K (2017). Bioactivity of the putative apelin proprotein expands the repertoire of apelin receptor ligands. Biochim Biophys Acta.

[10] Shin K, Pandey A, Liu XQ, Anini Y, Rainey JK (2013). Preferential apelin-13 production by the proprotein convertase PCSK3 is implicated in obesity. FEBS Open Bio.

[11] De Mota N (2004). Apelin, a potent diuretic neuropeptide counteracting vasopressin actions through inhibition of vasopressin neuron activity and vasopressin release. Proc Natl Acad Sci USA.

[12] Miettinen KH (2007). Utility of plasma apelin and other indices of cardiac dysfunction in the clinical assessment of patients with dilated cardiomyopathy. Regul Pept.

[13] Azizi M (2008). Reciprocal regulation of plasma apelin and vasopressin by osmotic stimuli. J Am Soc Nephrol.

[14] Zhen EY, Higgs RE, Gutierrez JA (2013). Pyroglutamyl apelin-13 identified as the major apelin isoform in human plasma. Anal Biochem.

[15] Sargent DF, Schwyzer R (1986). Membrane lipid phase as catalyst for peptide-receptor interactions. Proc Natl Acad Sci USA.

[16] Mandaliti W (2016). Thymosin α1 interacts with exposed phosphatidylserine in membrane models and in cells and uses serum albumin as a carrier. Biochemistry.

[17] Langelaan DN, Rainey JK (2009). Headgroup-dependent membrane catalysis of apelin-receptor interactions is likely. J Phys Chem B.

[18] Motta A, Andreotti G, Amodeo P, Strazzullo G, Castiglione Morelli MA (1998). Solution structure of human calcitonin in membrane-mimetic environment: the role of the amphipathic helix. Proteins.

[19] Lopes SC, Fedorov A, Castanho MA (2005). Lipidic membranes are potential “catalysts” in the ligand activity of the multifunctional pentapeptide neokyotorphin. Chembiochem.

[20] Contreras LM, de Almeida RF, Villalain J, Fedorov A, Prieto M (2001). Interaction of α-melanocyte stimulating hormone with binary phospholipid membranes: structural changes and relevance of phase behavior. Biophys J.

[21] Huang SK, Shin K, Sarker M, Rainey JK (2017). Apela exhibits isoform- and headgroup-dependent modulation of micelle binding, peptide conformation and dynamics. Biochim Biophys Acta.

[22] Langelaan DN, Rainey JK (2010). Membrane catalysis of peptide-receptor binding. Biochem Cell Biol.

[23] van Meer G, Voelker DR, Feigenson GW (2008). Membrane lipids: where they are and how they behave. Nat Rev Mol Cell Biol.

[24] Thomas S, Preda-Pais A, Casares S, Brumeanu TD (2004). Analysis of lipid rafts in T cells. Mol Immunol.

[25] Korade Z, Kenworthy AK (2008). Lipid rafts, cholesterol, and the brain. Neuropharmacology.

[26] Honke K (2013). Biosynthesis and biological function of sulfoglycolipids. Proc Jpn Acad Ser B Phys Biol Sci.

[27] Macaluso NJ, Glen RC (2010). Exploring the ‘RPRL’ motif of apelin-13 through molecular simulation and biological evaluation of cyclic peptide analogues. ChemMedChem.

[28] Macaluso NJ, Pitkin SL, Maguire JJ, Davenport AP, Glen RC (2011). Discovery of a competitive apelin receptor (APJ) antagonist. ChemMedChem.

[29] Murza A (2012). Elucidation of the structure-activity relationships of apelin: influence of unnatural amino acids on binding, signaling, and plasma stability. ChemMedChem.

[30] Juhl C (2016). Development of potent and metabolically stable APJ ligands with high therapeutic potential. ChemMedChem.

[31] Murza A (2017). Structure-activity relationship of novel macrocyclic biased apelin receptor agonists. Org Biomol Chem.

[32] Tatemoto K (1998). Isolation and characterization of a novel endogenous peptide ligand for the human APJ receptor. Biochem Biophys Res Commun.

[33] Habata Y (1999). Apelin, the natural ligand of the orphan receptor APJ, is abundantly secreted in the colostrum. Biochim Biophys Acta.

[34] Patterson RE, Weatherbee-Martin N, Rainey JK (2017). Pyrene-apelin conjugation modulates fluorophore- and peptide-micelle interactions. J Phys Chem B.

[35] Fan X (2003). Structural and functional study of the apelin-13 peptide, an endogenous ligand of the HIV-1 coreceptor, APJ. Biochemistry.

[36] Langelaan DN, Bebbington EM, Reddy T, Rainey JK (2009). Structural insight into G-protein coupled receptor binding by apelin. Biochemistry.

[37] Kelly SM, Jess TJ, Price NC (2005). How to study proteins by circular dichroism. Biochim Biophys Acta.

[38] Woody, R. W. In *Peptides, Polypeptides and Proteins* (John Wiley and Sons Inc. 1974).

[39] Wu DH, Chen AD, Johnson CS (1995). An improved diffusion-ordered spectroscopy experiment incorporating bipolar-gradient pulses. J Magn Reson Ser A.

[40] Cavanagh, J., Fairbrother, W. J., Palmer III, A. G., Rance, M. & Skelton, N. J. *Protein NMR spectroscopy, principles and practice*. 2nd edn, (Academic Press, 2006).

[41] Lipfert J, Columbus L, Chu VB, Lesley SA, Doniach S (2007). Size and shape of detergent micelles determined by small-angle X-ray scattering. J Phys Chem B.

[42] Toth G, Madarasz A (2006). Structure of BRIJ-35 nonionic surfactant in water: a reverse Monte Carlo study. Langmuir.

[43] Jönsson B, Wennerström H, Nilsson PG, Linse P (1986). Self-diffusion of small molecules in colloidal systems. Colloid Polym. Sci..

[44] Wishart DS, Sykes BD, Richards FM (1992). The chemical shift index: a fast and simple method for the assignment of protein secondary structure through NMR spectroscopy. Biochemistry.

[45] Cheung MS, Maguire ML, Stevens TJ, Broadhurst RW (2010). DANGLE: A Bayesian inferential method for predicting protein backbone dihedral angles and secondary structure. J Magn Reson.

[46] Bai Y, Milne JS, Mayne L, Englander SW (1993). Primary structure effects on peptide group hydrogen exchange. Proteins.

[47] Williamson MP (2013). Using chemical shift perturbation to characterise ligand binding. Prog Nucl Magn Reson Spectrosc.

[48] Kleckner IR, Foster MP (2011). An introduction to NMR-based approaches for measuring protein dynamics. Biochim Biophys Acta.

[49] Ma Y (2017). Structural basis for apelin control of the human apelin receptor. Structure.

[50] Nicolson GL (2014). The fluid-mosaic model of membrane structure: still relevant to understanding the structure, function and dynamics of biological membranes after more than 40 years. Biochim Biophys Acta.

[51] Gill SC, von Hippel PH (1989). Calculation of protein extinction coefficients from amino acid sequence data. Anal Biochem.

[52] Anachkov SE, Danov KD, Basheva ES, Kralchevsky PA, Ananthapadmanabhan KP (2012). Determination of the aggregation number and charge of ionic surfactant micelles from the stepwise thinning of foam films. Adv Colloid Interface Sci.

[53] Becher P (1961). Nonionic surface-active compounds IV. Micelle formation by polyoxyethylene alkanols and alkyl phenols in aqueous solution. J Colloid Sci.

[54] Palmer AG, Cavanagh J, Wright PE, Rance M (1991). Sensitivity improvement in proton-detected two-dimensional heteronuclear correlation NMR spectroscopy. J Magn Reson.

[55] Kay LE, Keifer P, Saarinen T (1992). Pure absorption gradient enhanced heteronuclear single quantum correlation spectroscopy with improved sensitivity. J Am Chem Soc.

[56] Schleucher J (1994). A general enhancement scheme in heteronuclear multidimensional NMR employing pulsed field gradients. J Biomol NMR.

[57] Grzesiek S, Bax A (1993). The importance of not saturating H_2_O in protein NMR - Application to sensitivity enhancement and NOE measurements. J Am Chem Soc.

[58] Stilbs P, Fourier-transform NMR (1982). pulsed-gradient spin-echo (FT-PGSE) self-diffusion measurements of solubilization equilibria in SDS solutions. J. Colloid Interface Sci.

[59] Delaglio F (1995). NMRPipe: a multidimensional spectral processing system based on UNIX pipes. J Biomol NMR.

[60] Wishart DS (1995). ^1^H, ^13^C and ^15^N chemical shift referencing in biomolecular NMR. J Biomol NMR.

[61] Vranken WF (2005). The CCPN data model for NMR spectroscopy: development of a software pipeline. Proteins.

[62] Schumann FH (2007). Combined chemical shift changes and amino acid specific chemical shift mapping of protein-protein interactions. J Biomol NMR.

[63] Lide, D. R. In CRC *Handbook of Chemistry and Physics* (ed D. R. Lide) Ch. 7, (CRC Press, Boca Raton, FL, 2008).

