# Supplementary material for: Apelin conformational and binding equilibria upon micelle interaction primarily depend on membrane-mimetic headgroup
Source: Sci Rep. 2017 Nov 13;7:15433. doi: 10.1038/s41598-017-14784-0 (PMC5684411; doi:10.1038/s41598-017-14784-0)
Supplement: Supplementary file 1 — Supplementary Information [file 41598_2017_14784_MOESM1_ESM.pdf]

Supplementary Information for:

Apelin conformational and binding equilibria upon micelle interaction primarily depend on membrane-mimetic headgroup

Kyungsoo Shin[a], Muzaddid Sarker[a], Shuya K. Huang[a], Jan K. Rainey*[,a,b]

[a] Department of Biochemistry & Molecular Biology, Dalhousie University, Halifax, Nova Scotia, Canada, B3H 4R2

[b] Department of Chemistry, Dalhousie University, Halifax, Nova Scotia, Canada, B3H 4R2

* To whom correspondence should be addressed. E-mail: jan.rainey@dal.ca.

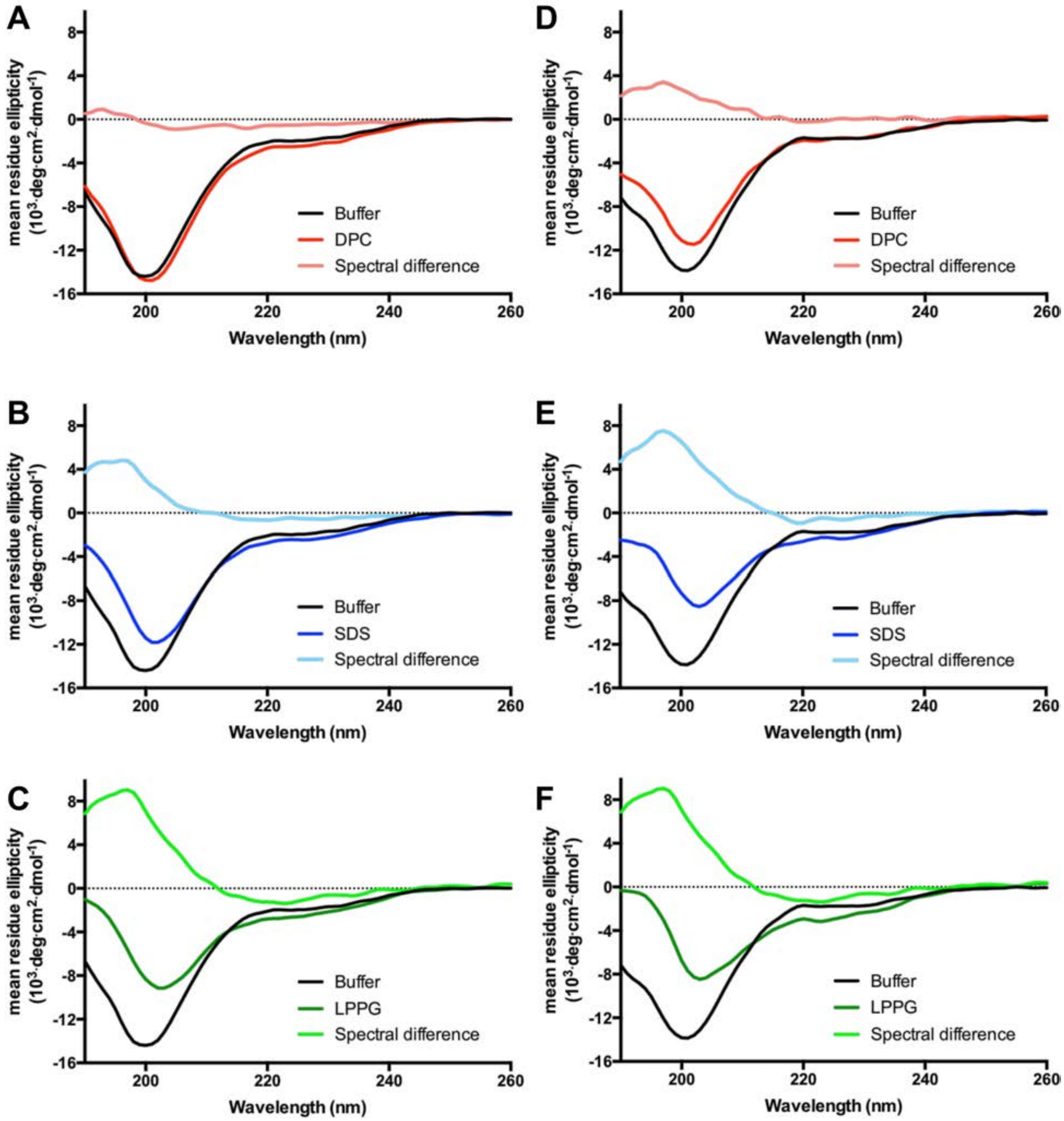

Supplementary figure S1: Characterization of apelin-micelle interaction by far-UV CD spectropolarimetry. Spectral difference for apelin-55 (A, B, C) and -36 (D, E, F) were acquired through subtraction of mean residue ellipticity in buffer from that in indicated micelle conditions.

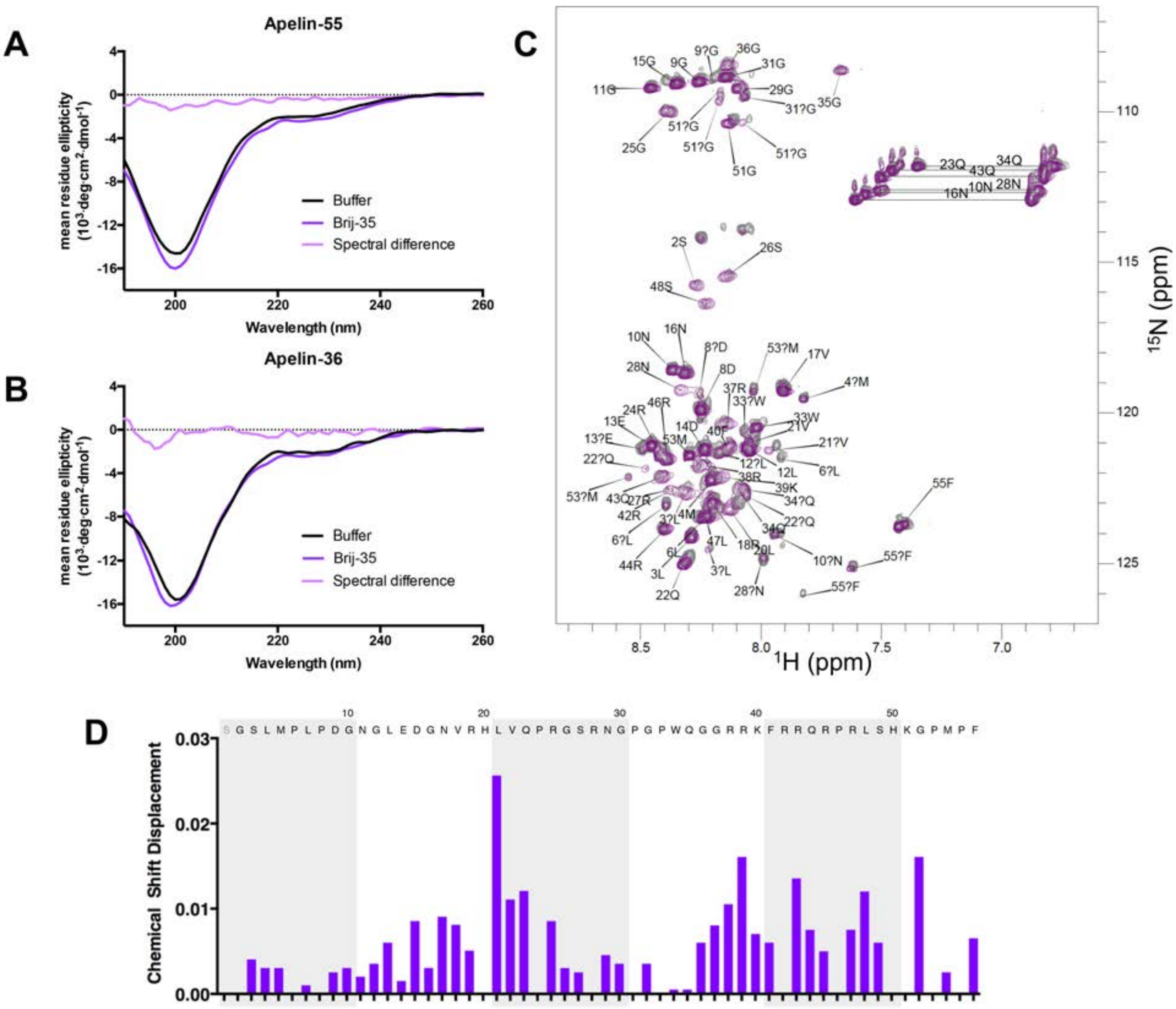

Supplementary figure S2: Summary of apelin-Brij-35 interaction. Far-UV CD spectra of A) apelin-55 and B) apelin-36 in the presence of Brij-35 micelles. C) Annotated ^{1}H-^{15}N HSQC spectrum of apelin-55 with Brij-35 overlaid on apelin-55 in buffer (re-plotted from Shin *et al.*[1]). Peak identities were inferred from the annotated ^{1}H and ^{15}N chemical shifts of apelin-55 in buffer. D) Euclidian combined chemical shift displacement for H_N and N of apelin-55 with Brij-35 relative to buffer.

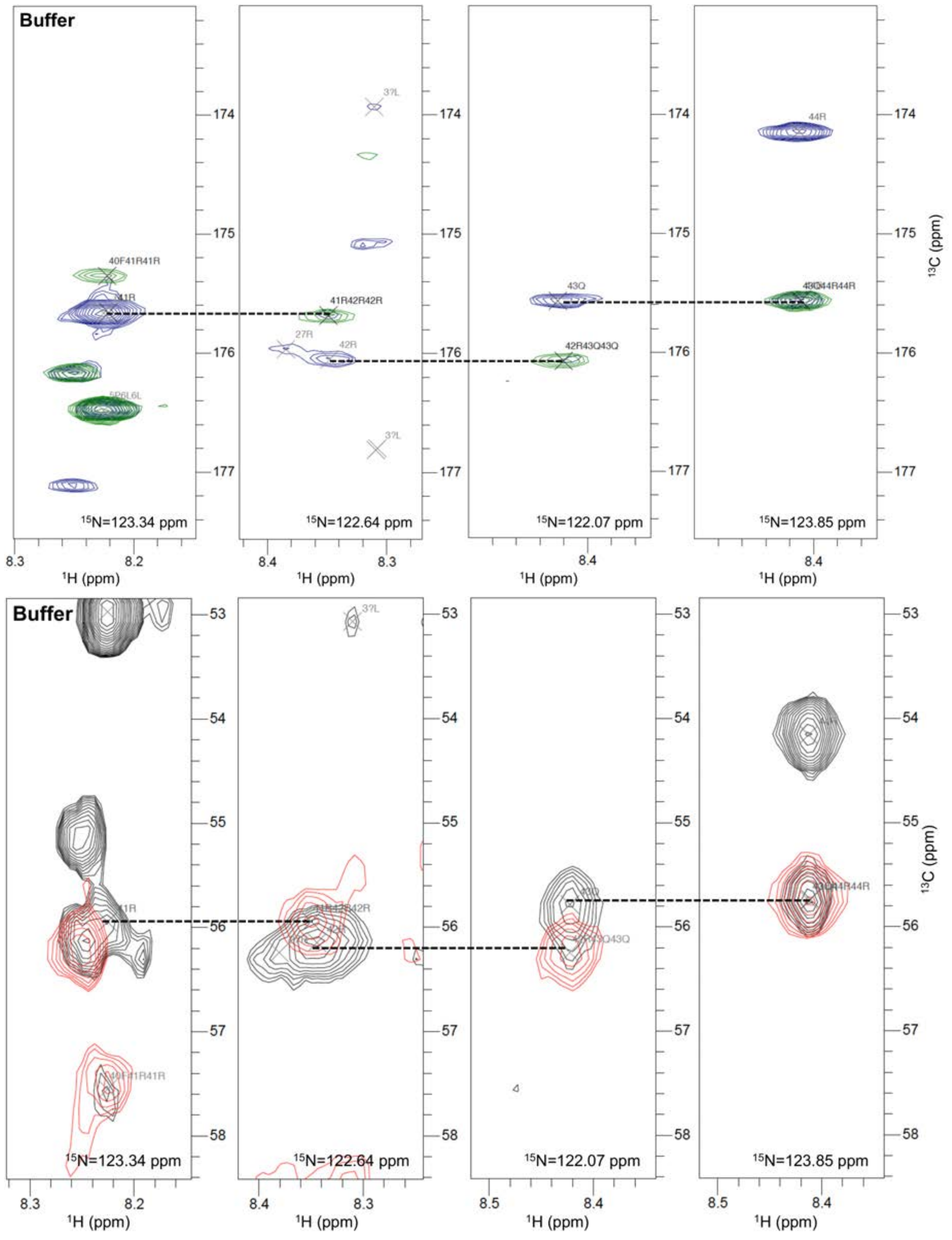
Buffer
40F41R41R
41R
41R42R42R
27R
42R
3?L
43Q
42R43Q43Q
44R
43Q44R44R
¹⁵N=123.34 ppm
¹⁵N=122.64 ppm
¹⁵N=122.07 ppm
¹⁵N=123.85 ppm
¹H (ppm)
¹³C (ppm)

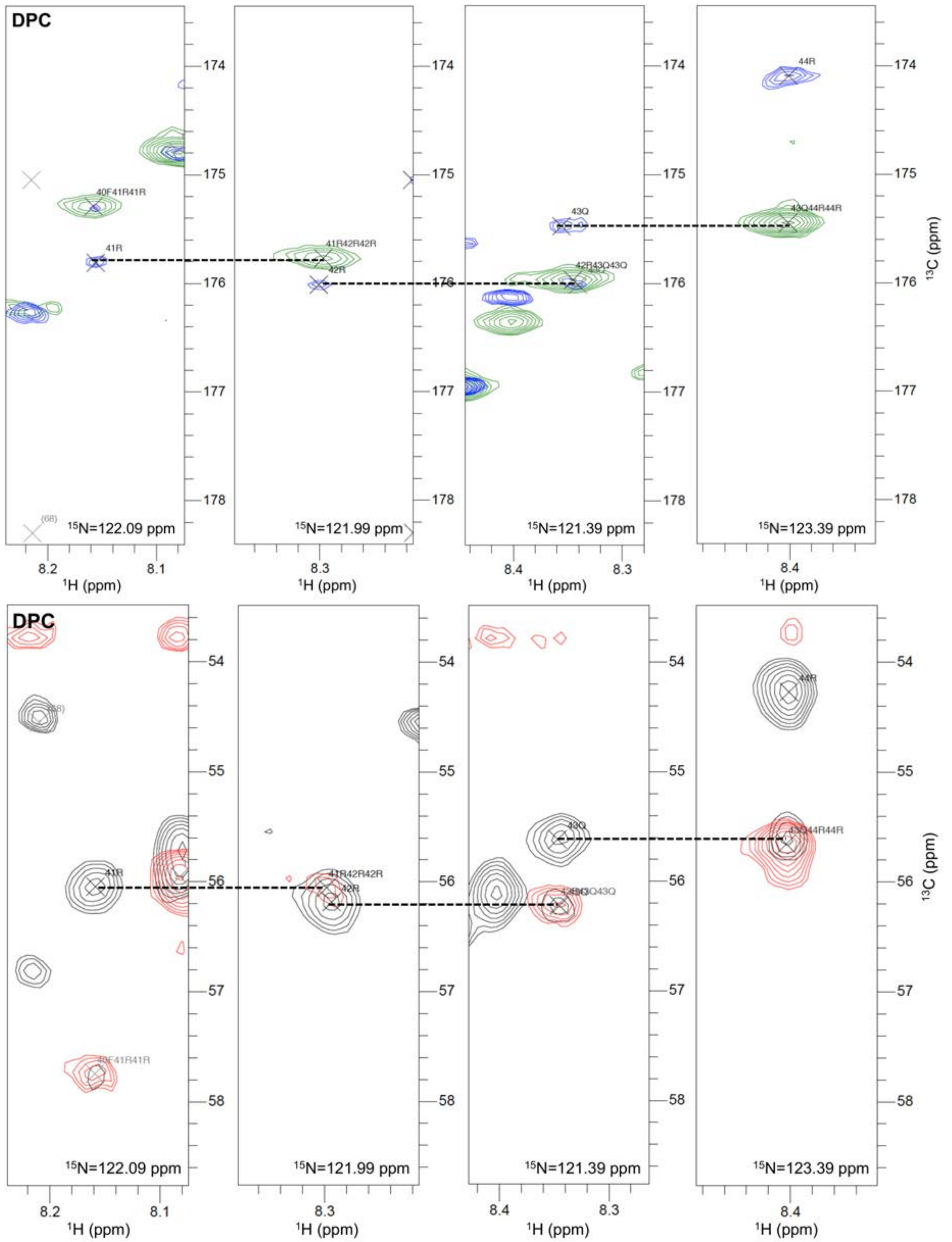
DPC
40F41R41R
41R
41R42R42R
42R
43Q
42R43Q43Q
44R
43Q44R44R
¹⁵N=122.09 ppm
¹⁵N=121.99 ppm
¹⁵N=121.39 ppm
¹⁵N=123.39 ppm
¹H (ppm)
¹³C (ppm)
DPC
41R
41R42R42R
42R
43Q
43Q43Q
44R
43Q44R44R
40F41R41R
¹⁵N=122.09 ppm
¹⁵N=121.99 ppm
¹⁵N=121.39 ppm
¹⁵N=123.39 ppm
¹H (ppm)
¹³C (ppm)

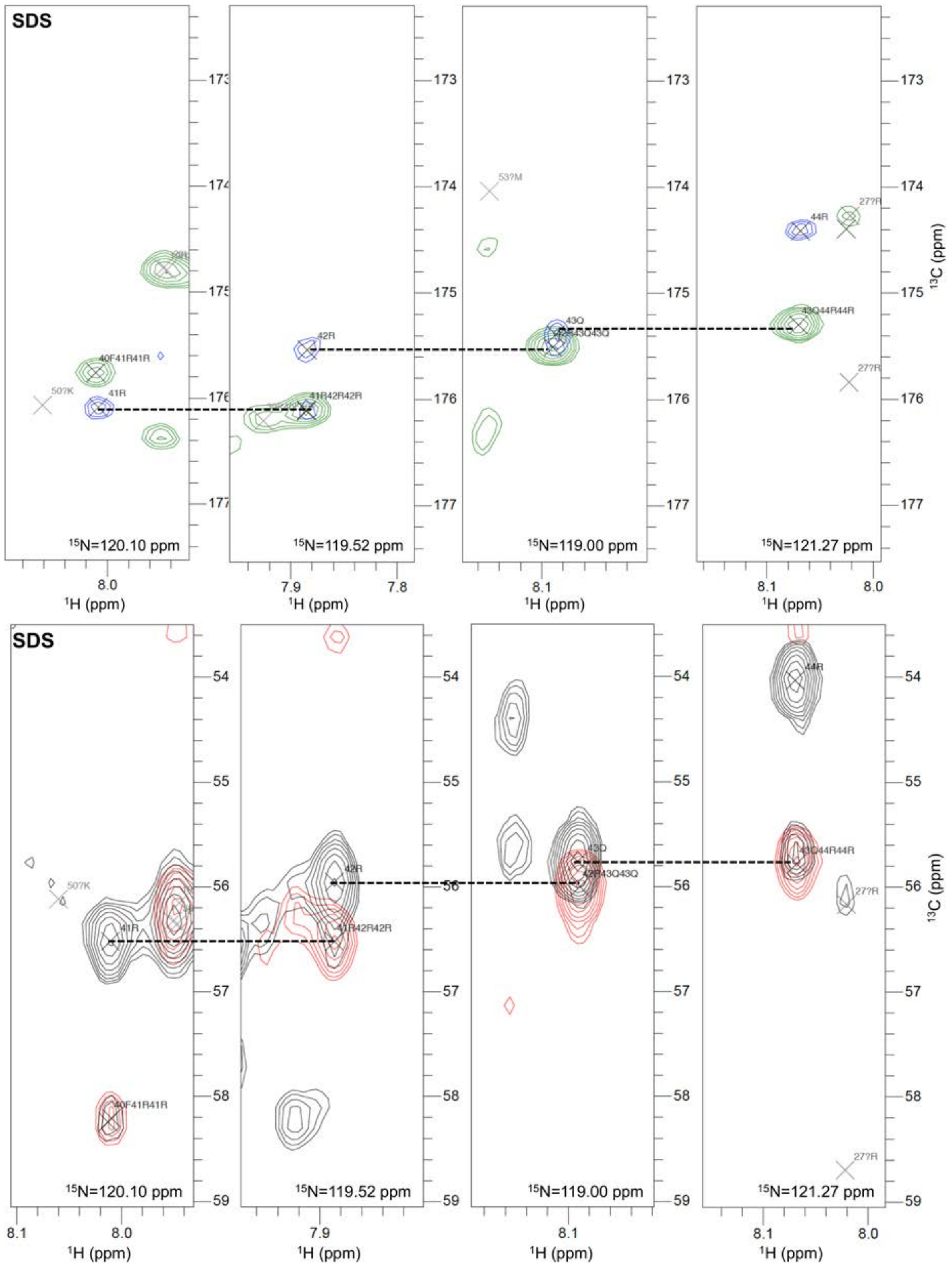
SDS
¹⁵N=120.10 ppm
¹⁵N=119.52 ppm
¹⁵N=119.00 ppm
¹⁵N=121.27 ppm
40F41R41R
41R
42R
41R42R42R
43Q
42R43Q43Q
44R
43Q44R44R
27?R
50?K
53?M
¹H (ppm)
¹³C (ppm)
SDS
¹⁵N=120.10 ppm
¹⁵N=119.52 ppm
¹⁵N=119.00 ppm
¹⁵N=121.27 ppm
41R
40F41R41R
42R
41R42R42R
43Q
42R43Q43Q
44R
43Q44R44R
27?R
50?K
¹H (ppm)
¹³C (ppm)

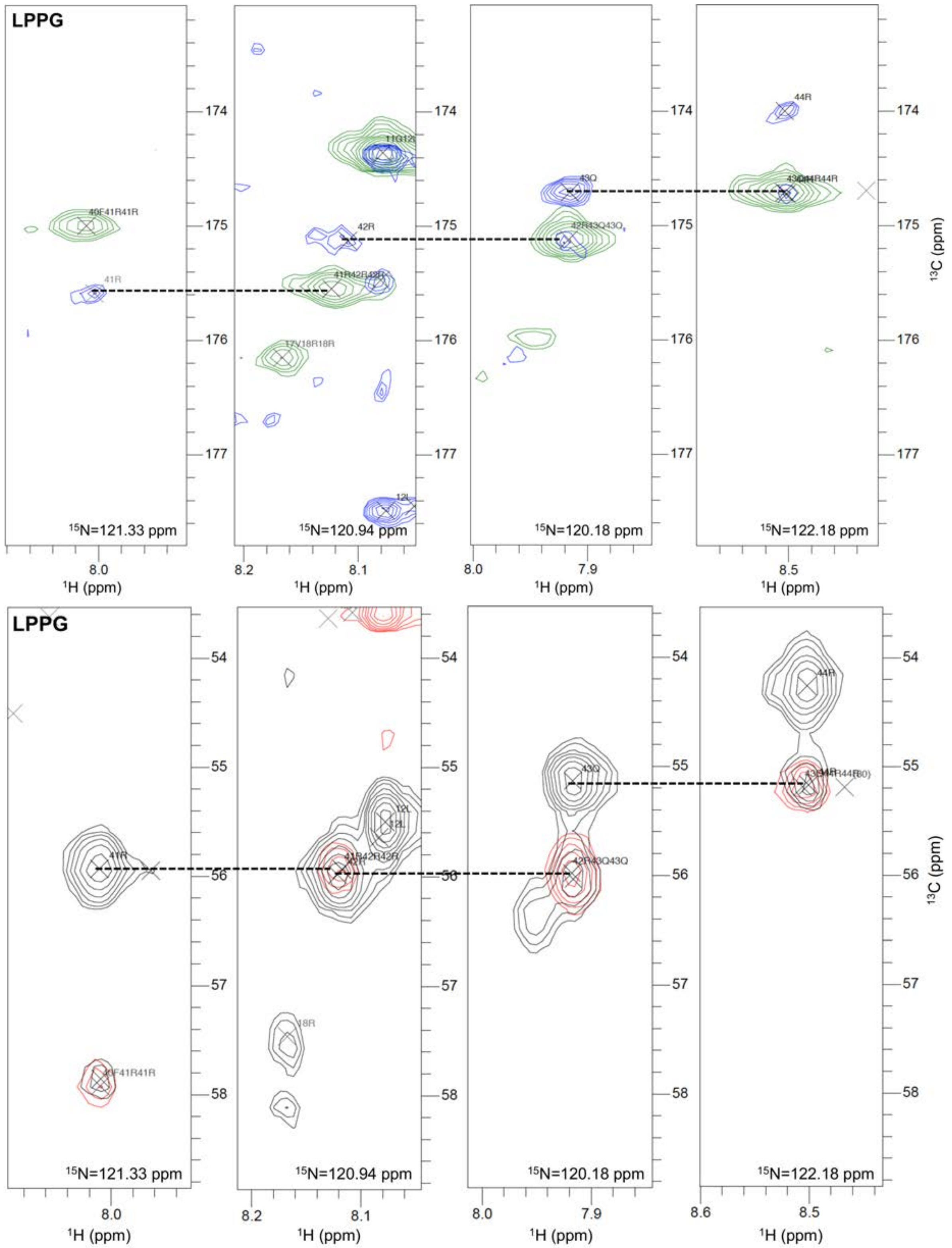

Supplementary figure S3 (*pages 3-7*): Main-chain directed backbone assignments for 41R-44R segment of apelin-55 demonstrated using HNCaCO (blue), HNCO (green), HNCA (black), and HNCoCA (red) in indicated conditions.

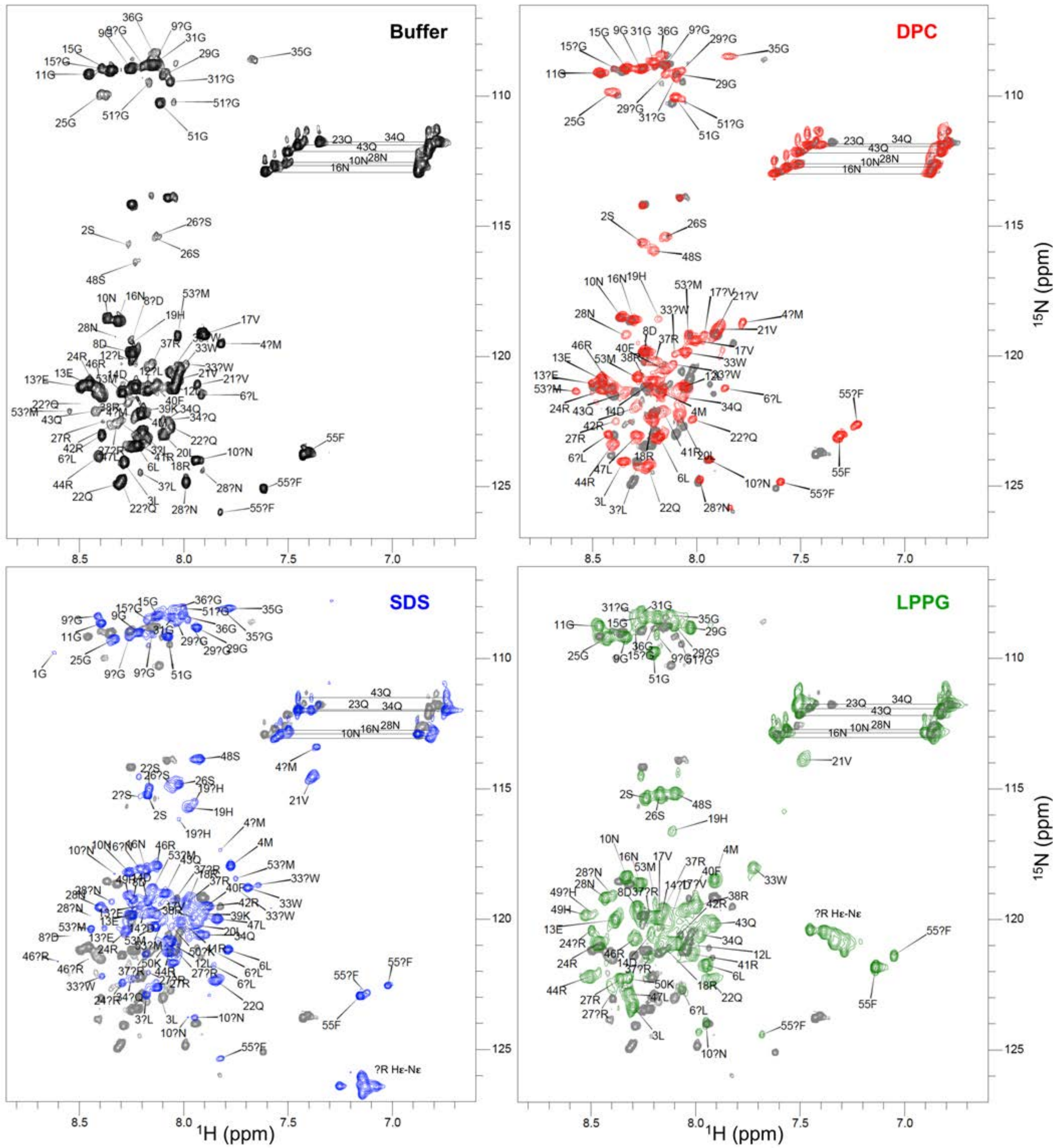

Supplementary figure S4: ^{1}H-^{15}N HSQC spectra of apelin-55 in indicated micellar condition with cross-peaks annotated by number in the sequence and single-letter amino acid code. Additional resonance assignment from alternative conformations are differentiated by a question mark. The assigned spectrum of apelin-55 in buffer is re-plotted from Shin *et al*.[1]. Spectra of apelin-55 in micellar conditions (coloured) are overlaid on the spectrum in buffer (grey).

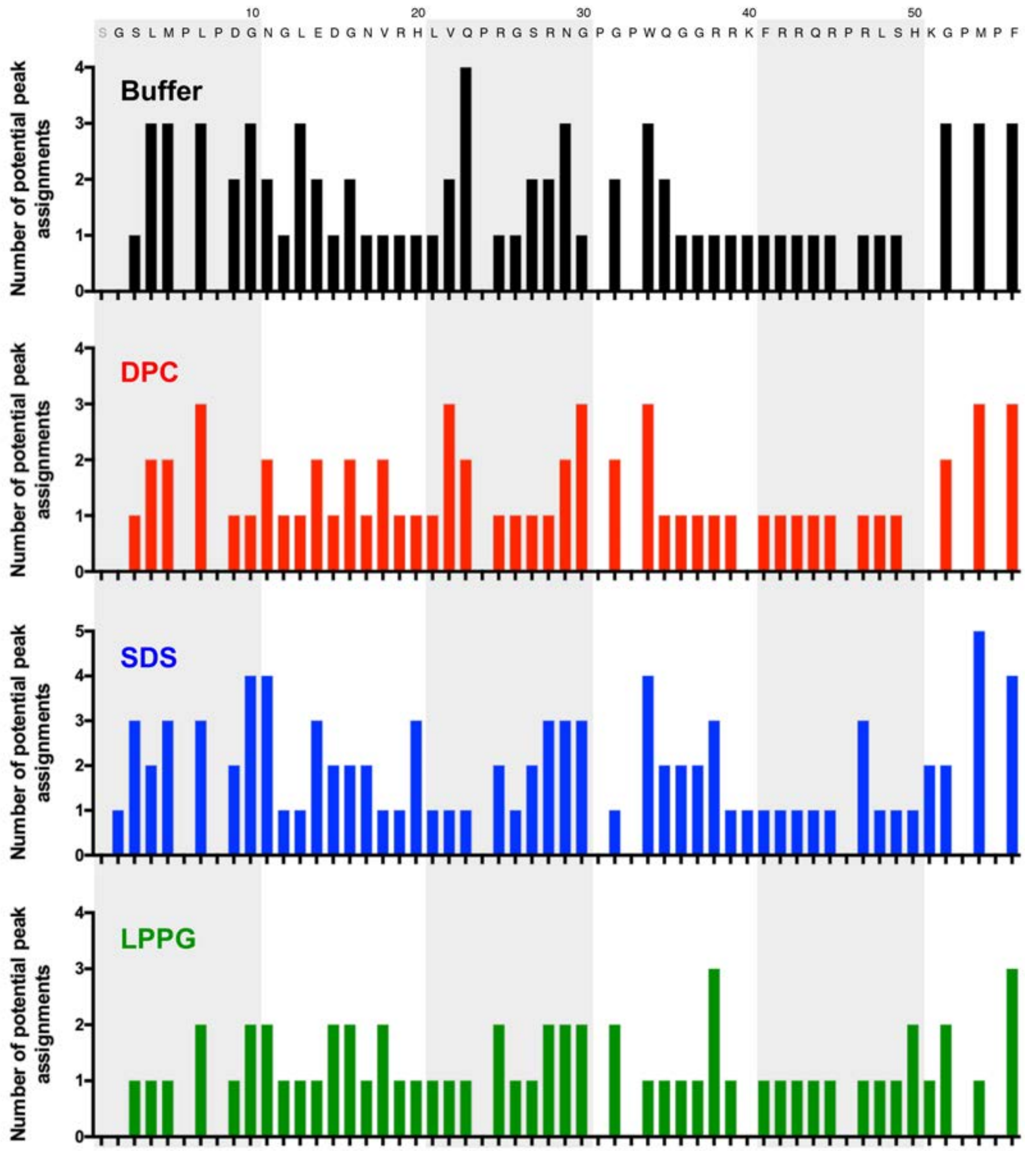

Supplementary figure S5: Number of potential spin system assignments per residue of apelin-55 in indicated micelle condition. For comparison, the number of potential assignments observed for apelin-55 in buffer are re-plotted from Shin *et al*.[1].

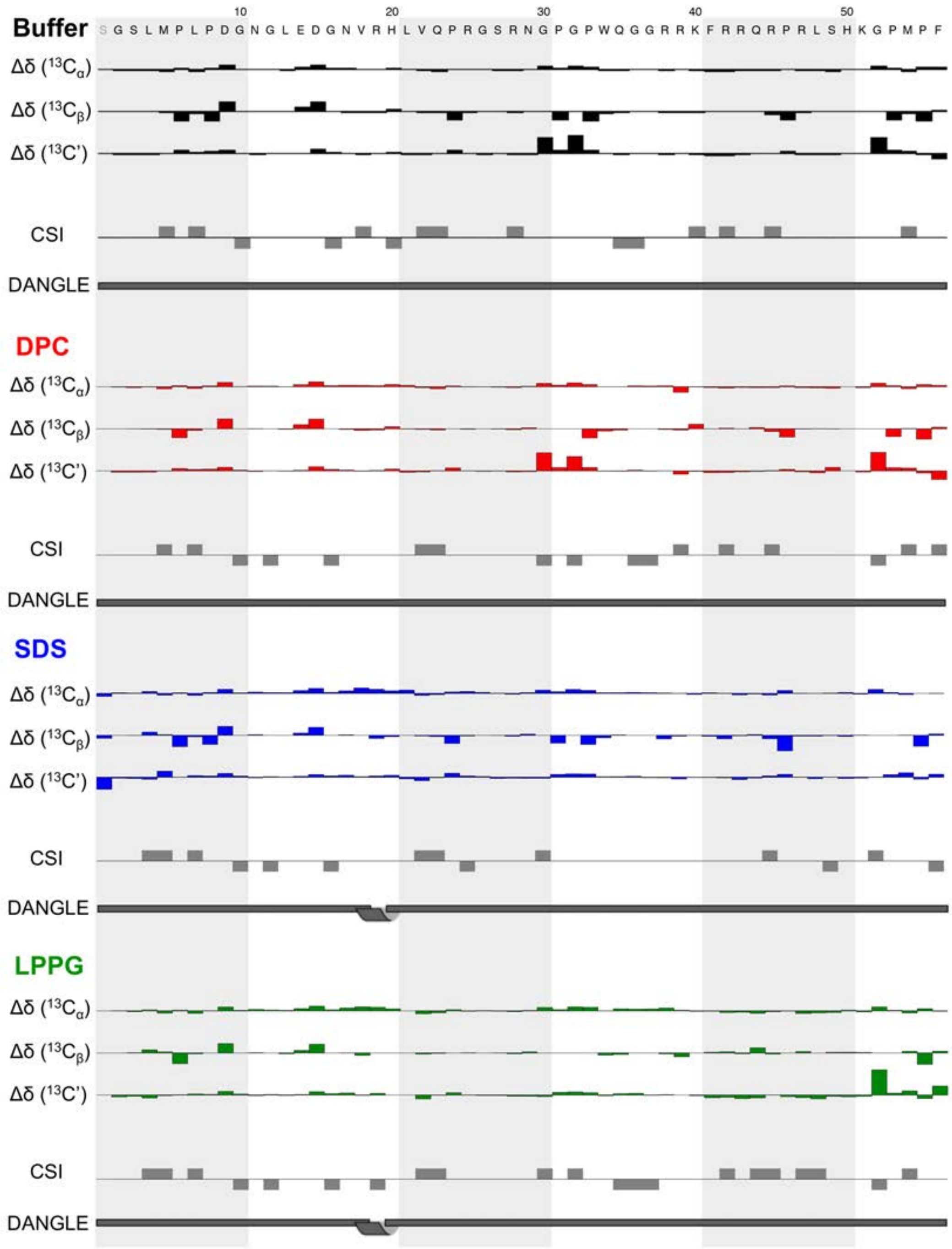

Supplementary figure S6: Secondary chemical shift (Δδ) for given nucleus, CSI, and DANGLE for apelin-55 in indicated micelle condition. Δδ and CSI for apelin-55 in buffer are re-plotted from Shin *et al.*[1] for comparison. DANGLE for apelin-55 in buffer is based on the chemical shifts previously reported in Shin *et al.*[1].

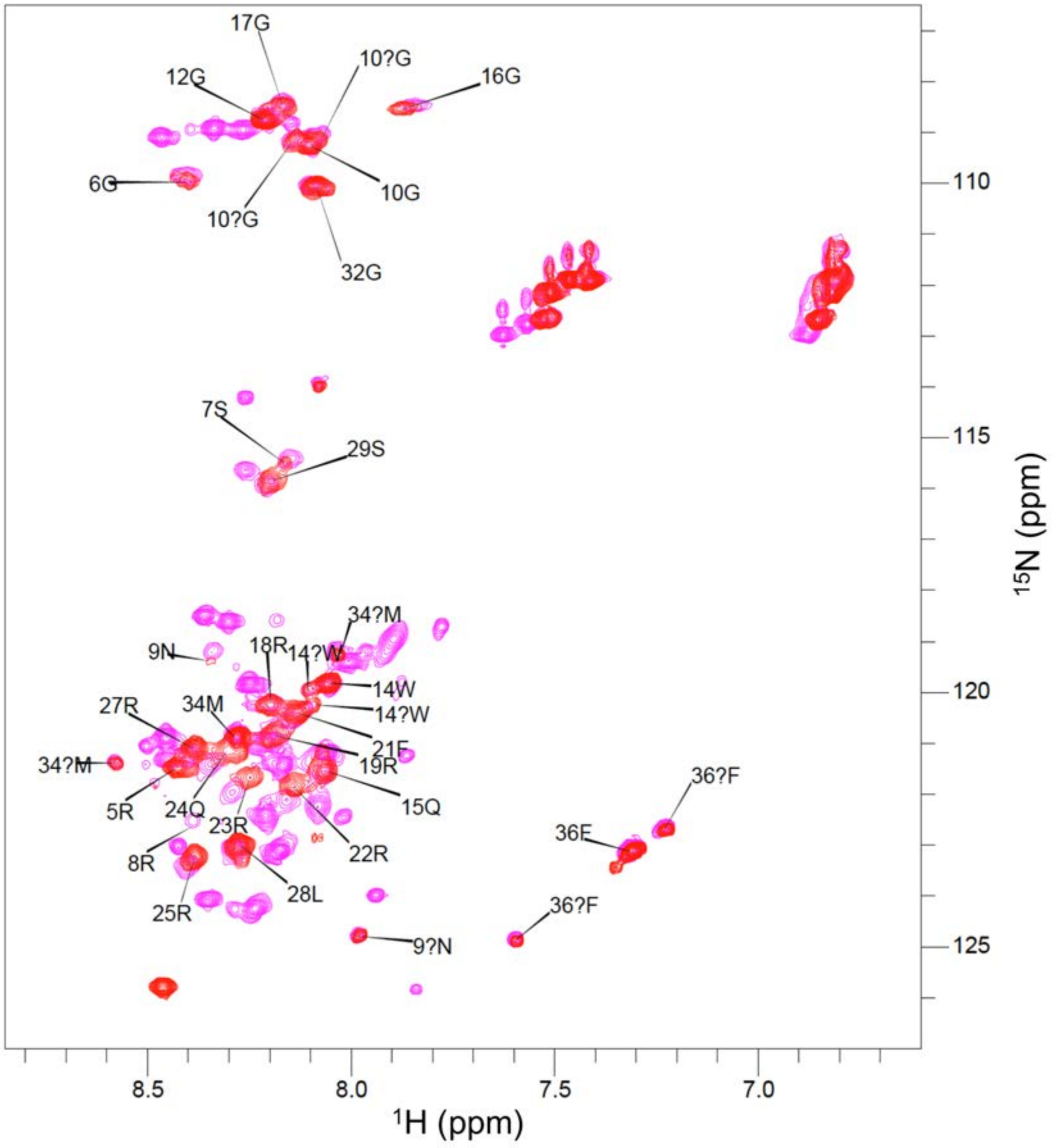

Supplementary figure S7: Annotated ^{1}H-^{15}N HSQC spectrum of apelin-36 (red) overlaid on that of apelin-55 (magenta) in the presence of DPC micelles. Peak identities for apelin-36 were inferred from assigned chemical shifts of apelin-55 in the same micelle condition.

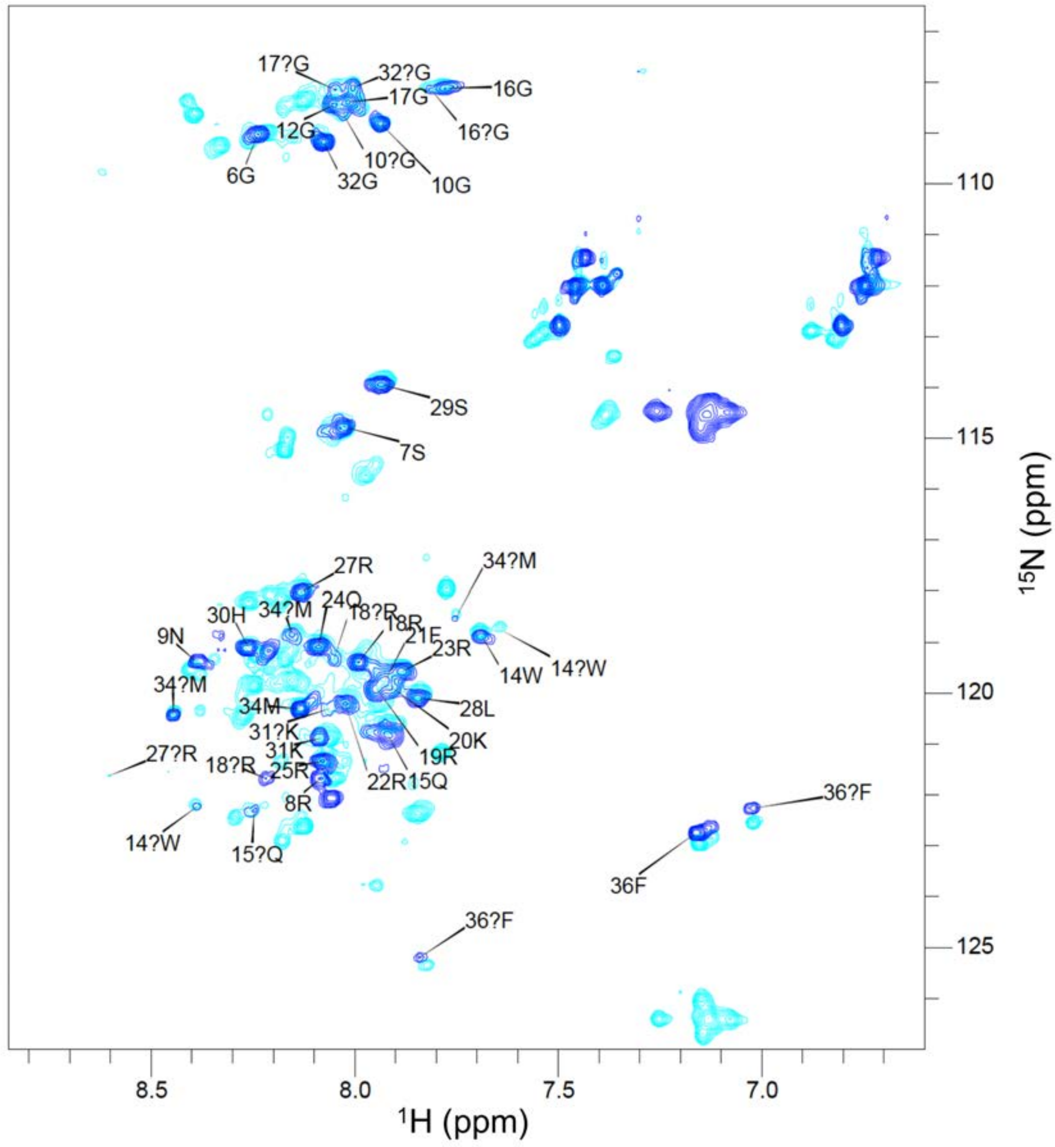

Supplementary figure S8: Annotated ^{1}H-^{15}N HSQC spectrum of apelin-36 (blue) overlaid on that of apelin-55 (cyan) in the presence of SDS micelles. Peak identities for apelin-36 were inferred from assigned chemical shifts of apelin-55 in the same micelle condition.

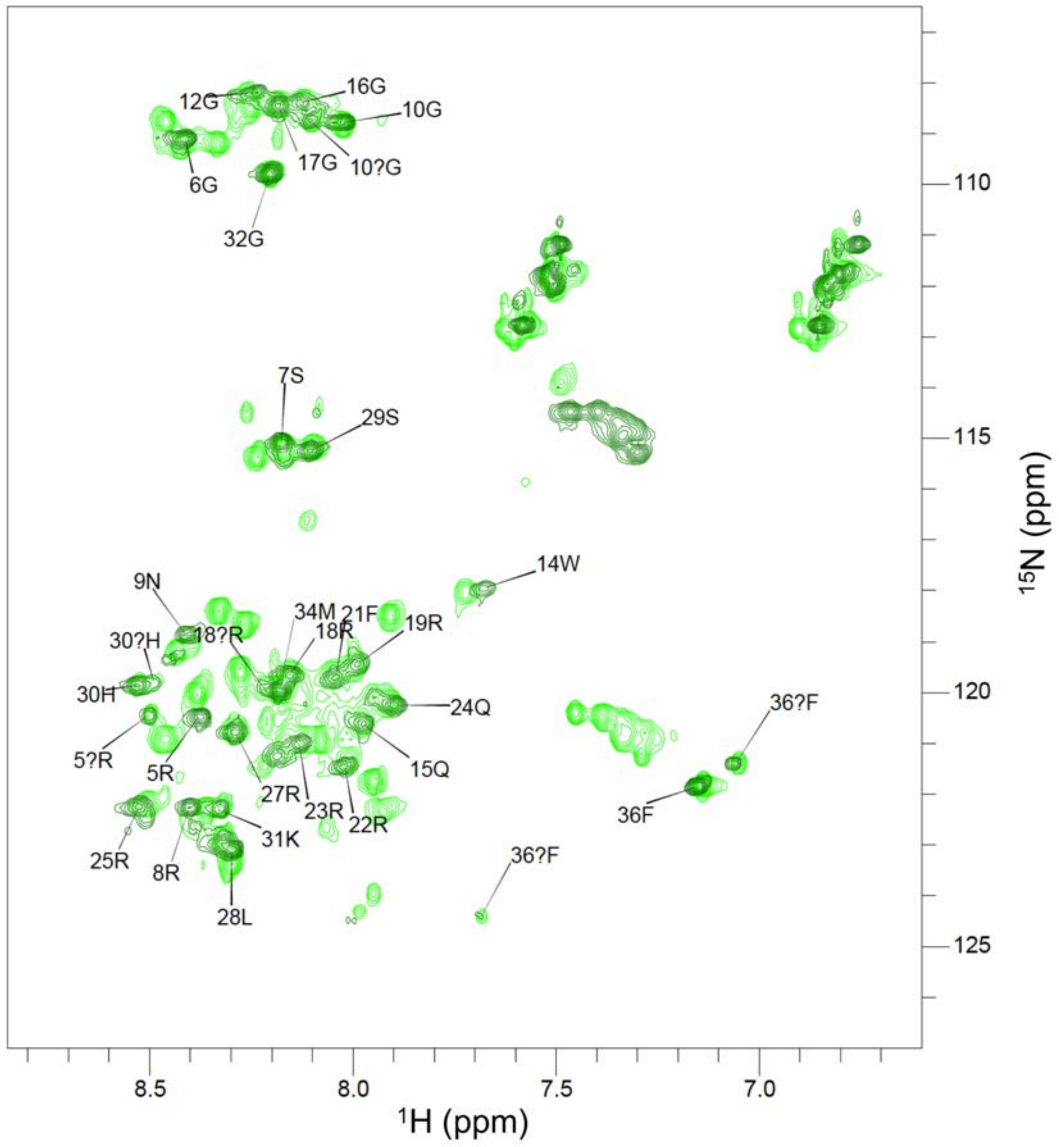

Supplementary figure S9: Annotated ^{1}H-^{15}N HSQC spectrum of apelin-36 (green) overlaid on that of apelin-55 (lime) in the presence of LPPG micelles. Peak identities for apelin-36 were inferred from assigned chemical shifts of apelin-55 in the same micelle condition.

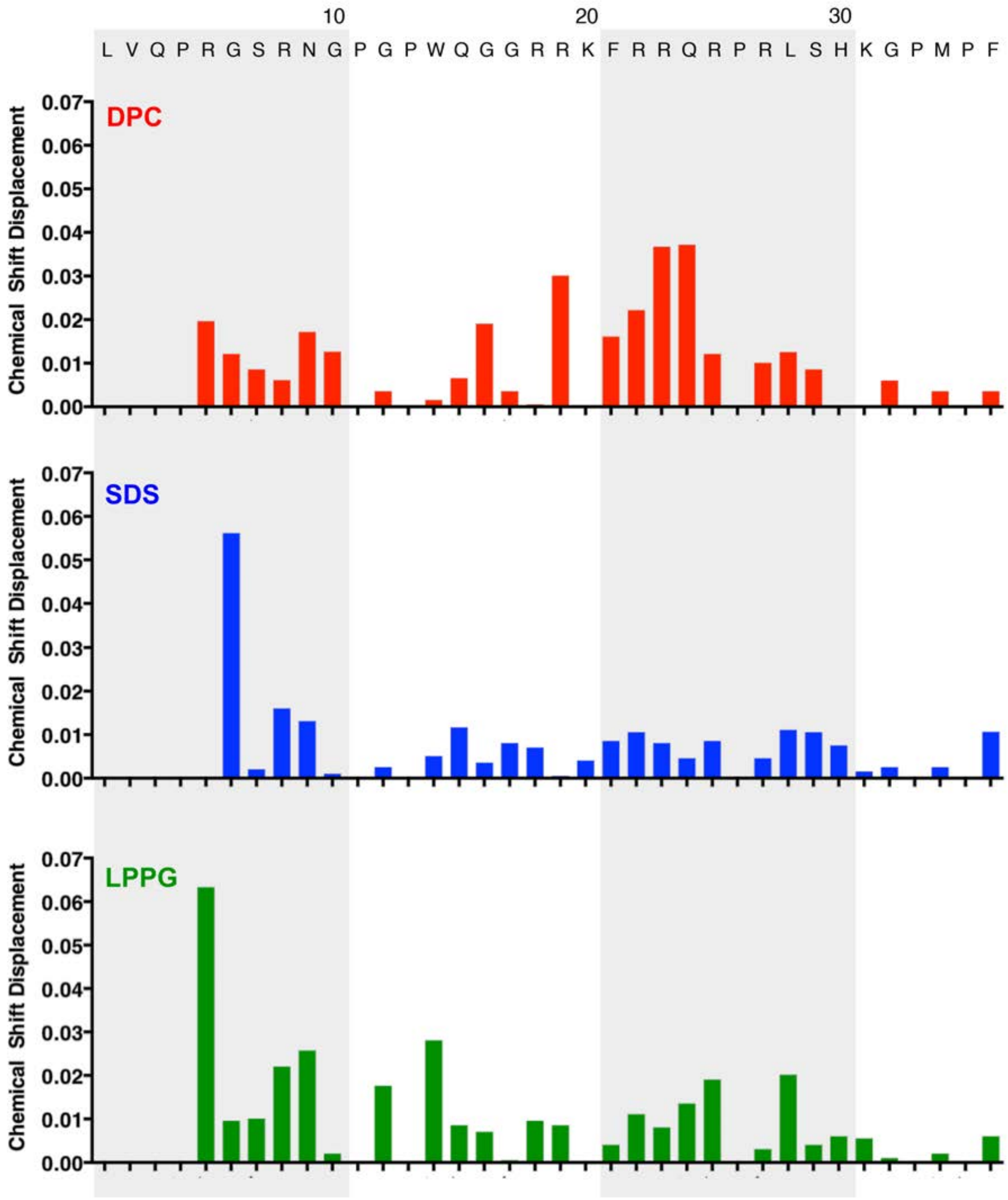

Supplementary figure S10: Euclidian combined chemical shift displacement for indicated residue of apelin-36 relative to the corresponding residue in apelin-55 based upon H_N and N chemical shifts in each of the indicated micelle conditions.

Supplementary Table S1: Viscosity-corrected diffusion coefficients (D_{ob}) of free micelles, free peptide and peptide in the presence of each micelle for apelin-55 and apelin-36 as determined by DOSY. Fraction of binding (f_b) was determined in each case using Eq. 3. Viscosity (η) and the optimized gradient length (δ) and diffusion time (Δ) are also detailed. (Values are presented as mean ± SEM.)

Experimental set	Component	Nucleus detected#	# of gradient steps	Gradient amplitude#	Δ (ms)	δ (ms)	η (mPa·s)	D_{ob} (10^{-10} m^2 s^{-1})	f_b
Apelin-55	Free apelin-55	^{15}N/^{1}H	9/16	^{15}N: 2-98%<br>^{1}H: 2-95%	100	1.25/2	0.715 ± 0.003	1.850 ± 0.006	NA
	Apelin-55 with DPC micelle	^{15}N/^{1}H	9/16	^{15}N: 2-98%<br>^{1}H: 2-95%	100	1.75/3	0.738 ± 0.002	1.570 ± 0.009	0.266 ± 0.11
	Apelin-55 with SDS micelle	^{15}N/^{1}H	9/16	^{15}N: 2-98%<br>^{1}H: 2-95%	100	1.75/3	0.738 ± 0.001	1.070 ± 0.004	0.731 ± 0.08
	Apelin-55 with LPPG micelle	^{15}N/^{1}H	9/16	^{15}N: 2-98%<br>^{1}H: 2-95%	100	1.75/3	0.780 ± 0.002	0.796 ± 0.006	0.835 ± 0.10
	Apelin-55 with Brij-35 micelle	^{15}N	13	2-98%	100	2	NA	1.623 ± 0.001*	0.167 ± 0.05
Apelin-36	Free apelin-36	^{15}N	13	2-98%	100	1.25	0.711 ± 0.006	2.780 ± 0.006	NA
	Apelin-36 with DPC micelle	^{15}N	13	2-98%	100	1.25	0.756 ± 0.001	2.640 ± 0.003	0.076 ± 0.03
	Apelin-36 with SDS micelle	^{15}N	13	2-98%	100	1.75	0.739 ± 0.007	1.420 ± 0.000	0.794 ± 0.05
	Apelin-36 with LPPG micelle	^{15}N	13	2-98%	100	2.25	0.896 ± 0.002	1.300 ± 0.001	0.720 ± 0.04
Control	No protein/micelle	NA	NA	NA	NA	NA	0.712 ± 0.002	NA	NA
	Free DPC micelle	^{31}P	16	2-95%	300	4	0.746 ± 0.004	1.306 ± 0.001	NA
	Free SDS micelle	^{1}H	16	2-95%	100	3	0.753 ± 0.001	1.337 ± 0.004	NA
	Free LPPG micelle	^{31}P	16	2-95%	500	4	0.884 ± 0.003	0.817 ± 0.001	NA
	Free Brij-35 micelle	^{1}H	16	2-95%	150	4	NA	0.633 ± 0.002*	NA

^{15}N symbol represent detection of amide protons and use of 1D ^{1}H-^{15}N HSQC experiments with increasing diffusion measurement gradient amplitude (pulse program detailed in Methods). * Apelin-55 with Brij-35 and Free Brij-35 D_{ob} values were viscosity corrected by indirect mechanism using D_{ob} of water molecules of No protein/micelle control (37.6 ± 0.1 x 10^{-10} m^2 s^{-1}) to the water molecules with Brij-35 micelles (No protein control: 37.0 ± 0.05 x 10^{-10} m^2 s^{-1}, with protein: 37.0 ± 0.1 x 10^{-10} m^2 s^{-1}).

Supplementary Table S2: H_N, H_δ, H_ε, N, C’, C_α, C_β chemical shifts (ppm) of apelin-55 with DPC micelles.

Residue	H_N	N	C’	C_α	C_β	H_δ	H_ε	Comments
**0Ser**	**-**	**-**	**-**	**-**	**-**	**-**	**-**	
**1Gly**	**-**	**-**	**173.8**	**45.31**	**-**	**-**	**-**	
**2Ser**	**8.26**	**115.63**	**174.36**	**58.27**	**64.05**	**-**	**-**	**-**
3?Leu #1	8.29	124.27	175.54	55.46	-	-	-	No visible Cβ peak
**3Leu**	**8.35**	**124.07**	**176.8**	**55.41**	**42.39**	**-**	**-**	**-**
4?Met #1	7.78	118.72	174.65	52.89	34.11	-	-	-
**4Met**	**8.17**	**121.43**	**173.89**	**53.11**	**32.79**	**-**	**-**	**-**
5?Pro #1	-	-	175.58	63.27	-	-	-	Coming from 6?Leu #1
5?Pro #2	-	-	175.75	62.56	34.40	-	-	Coming from 6?Leu #2
**5Pro**	**-**	**-**	**176.3**	**62.98**	**31.98**	**-**	**-**	**Coming from 6Leu**
6?Leu #1	7.86	121.25	175.70	52.78	43.13	-	-	-
6?Leu #2	8.42	123.05	175.69	53.47	41.64	-	-	-
**6Leu**	**8.18**	**123.11**	**175.62**	**52.92**	**41.96**	**-**	**-**	**-**
**7Pro**	**-**	**-**	**176.55**	**63.35**	**-**	**-**	**-**	**Coming from 8Asp**
**8Asp**	**8.25**	**119.84**	**176.82**	**54.34**	**41.44**	**-**	**-**	**-**
**9Gly**	**8.27**	**108.92**	**174.29**	**45.7**	**-**	**-**	**-**	**-**
10?Asn #1	7.94	124.00	173.37	54.90	41.06	-	-	-
**10Asn**	**8.36**	**118.47**	**175.75**	**53.50**	**39.14**	**6.86, 7.57**	**-**	**-**
**11Gly**	**8.47**	**109.08**	**174.23**	**45.68**	**-**	**-**	**-**	**-**
**12Leu**	**8.06**	**121.17**	**177.54**	**55.34**	**42.32**	**-**	**-**	**-**

13?Glu #1	8.50	121.03	176.26	56.77	30.12	-	-	-
**13Glu**	**8.45**	**120.88**	**176.26**	**56.78**	**30.19**	**-**	**-**	**-**
**14Asp**	**8.21**	**120.97**	**176.9**	**54.55**	**41.41**	**-**	**-**	**-**
15?Gly #1	8.39	108.91	175.37	45.72	-	-	-	-
**15Gly**	**8.34**	**108.89**	**174.36**	**45.90**	**-**	**-**	**-**	**-**
**16Asn**	**8.30**	**118.58**	**175.65**	**53.58**	**38.92**	**6.88, 7.63**	**-**	**-**
17?Val #1	7.96	119.22	176.22	63.03	32.32	-	-	-
**17Val**	**8.00**	**119.39**	**176.22**	**63.15**	**32.33**	**-**	**-**	**-**
**18Arg**	**8.21**	**122.41**	**176.24**	**56.70**	**30.56**	**-**	**-**	**-**
**19His**	**8.18**	**118.57**	**174.77**	**55.93**	**29.80**	**-**	**-**	**-**
**20Leu**	**8.08**	**122.22**	**176.88**	**55.71**	**42.41**	**-**	**-**	**-**
21?Val #1	7.88	119.88	174.91	62.27	-	-	-	No visible Cβ peak
21?Val #2	7.91	119.19	175.57	62.27	32.69	-	-	-
**21Val**	**7.90**	**118.96**	**175.52**	**62.08**	**32.66**	**-**	**-**	**-**
22?Gln #1	8.02	122.44	174.02	53.31	30.23	-	-	-
**22Gln**	**8.24**	**124.24**	**173.77**	**53.57**	**29.16**	**-**	**6.82, 7.47**	**-**
**23Pro**	**-**	**-**	**176.96**	**63.24**	**-**	**-**	**-**	**Coming from 24Arg**
**24Arg**	**8.45**	**121.28**	**176.94**	**56.45**	**30.8**	**-**	**-**	**-**
**25Gly**	**8.41**	**109.84**	**174.1**	**45.31**	**-**	**-**	**-**	**-**
**26Ser**	**8.15**	**115.44**	**174.74**	**58.43**	**64.00**	**-**	**-**	**-**
**27Arg**	**8.39**	**122.52**	**175.96**	**56.31**	**30.66**	**-**	**-**	**-**
28?Asn #1	7.98	124.77	173.30	54.85	40.73	-	-	-

**28Asn**	**8.34**	**119.14**	**175.14**	**53.25**	**39.38**	**6.85,7.53**	**-**	**-**
29?Gly #1	8.15	109.17	177.87	44.77	-	-	-	-
29?Gly #2	8.07	109.03	177.93	44.46	-	-	-	-
**29Gly**	**8.09**	**109.09**	**177.65**	**44.70**	**-**	**-**	**-**	**-**
30?Pro #1	-	-	177.04	63.37	-	-	-	Coming from 31?Gly #1<br>No visible Cβ peak
**30Pro**	**-**	**-**	**177.27**	**63.30**	**31.89**	**-**	**-**	**Coming from 31Gly**
31?Gly #1	8.10	109.23	177.98	43.35	-	-	-	-
**31Gly**	**8.21**	**108.69**	**176.25**	**44.62**	**-**	**-**	**-**	**-**
32?Pro #1	-	-	176.84	63.43	-	-	-	Coming from 33?Trp #1<br>No visible Cβ peak
32?Pro #2	-	-	176.91	63.52	-	-	-	Coming from 33?Trp #2<br>No visible Cβ peak
**32Pro**	**-**	**-**	**176.86**	**63.56**	**31.89**	**-**	**-**	**Coming from 33Trp**
33?Trp #1	8.11	120.27	176.39	57.61	29.21	-	-	-
33?Trp #2	8.11	119.98	176.39	57.56	29.06	-	-	-
**33Trp**	**8.06**	**119.84**	**176.46**	**57.56**	**29.05**	**-**	**10.35**	**-**
**34Gln**	**8.07**	**121.56**	**176.37**	**56.28**	**29.14**	**-**	**6.80, 7.41**	**-**
**35Gly**	**7.84**	**108.45**	**174.69**	**45.63**	**-**	**-**	**-**	**-**
**36Gly**	**8.17**	**108.42**	**174.25**	**45.55**	**-**	**-**	**-**	**-**
**37Arg**	**8.20**	**120.26**	**176.55**	**56.69**	**30.73**	**-**	**-**	**-**
**38Arg**	**8.24**	**120.94**	**175.08**	**54.52**	**30.55**	**-**	**-**	**-**

**39Lys**	**-**	**-**	**176.14**	**56.41**	**34.66**	**-**	**-**	**Coming from 40Phe**
**40Phe**	**8.15**	**120.52**	**175.3**	**57.77**	**39.76**	**-**	**-**	**-**
**41Arg**	**8.16**	**122.08**	**175.79**	**56.05**	**31.11**	**-**	**-**	**-**
**42Arg**	**8.29**	**121.99**	**175.99**	**56.2**	**30.94**	**-**	**-**	**-**
**43Gln**	**8.35**	**121.39**	**175.46**	**55.64**	**29.92**	**-**	**6.82, 7.51**	**-**
**44Arg**	**8.40**	**123.39**	**174.09**	**54.27**	**30.07**	**-**	**-**	**-**
**45Pro**	**-**	**-**	**176.34**	**63.19**	**32.18**	**-**	**-**	**Coming from 46Arg**
**46Arg**	**8.40**	**121.21**	**176.11**	**56.11**	**30.86**	**-**	**-**	**-**
**47Leu**	**8.29**	**123.06**	**176.9**	**55.02**	**42.5**	**-**	**-**	**-**
**48Ser**	**8.21**	**115.92**	**175.6**	**58.15**	**64.02**	**-**	**-**	**-**
**49His**	**-**	**-**	**-**	**-**	**-**	**-**	**-**	-
50?Lys #1	-	-	176.24	56.19	-	-	-	Coming from 51?Gly #1<br>No assignable Cβ peak
**50Lys**	**-**	**-**	**176.29**	**56.21**	**-**	**-**	**-**	**Coming from 51Gly**<br>**No assignable Cβ peak**
51?Gly #1	8.07	110.16	177.28	44.47	-	-	-	-
**51Gly**	**8.10**	**110.02**	**177.58**	**44.49**	**-**	**-**	**-**	**-**
52?Pro #1	-	-	175.96	63.16	-	-	-	Coming from 53?Met #1<br>No visible Cβ peak
52?Pro #2	-	-	176.42	62.74	34.31	-	-	Coming from 53?Met #2
**52Pro**	**-**	**-**	**176.82**	**63.23**	**32.34**	**-**	**-**	**Coming from 53Met**
53?Met #1	8.04	119.20	174.65	53.40	33.91	-	-	-

53?Met #2	8.58	121.34	174.94	53.41	32.19	-	-	-
**53Met**	**8.28**	**120.84**	**174.79**	**53.07**	**33.02**	**-**	**-**	**-**
54?Pro #1	-	-	174.73	63.33	34.19	-	-	Coming from 55?Phe #1
54?Pro #2	-	-	174.74	63.47	31.32	-	-	Coming from 55?Phe #2
**54Pro**	**-**	**-**	**175.02**	**63.44**	**31.51**	**-**	**-**	**Coming from 55Phe**
55?Phe #1	7.60	124.83	173.79	58.79	39.70	-	-	-
55?Phe #2	7.23	122.65	173.59	58.40	40.23	-	-	-
**55Phe**	**7.31**	**123.06**	**173.75**	**58.56**	**40.24**	**-**	**-**	**-**

Supplementary Table S3: H_N, H_δ, H_ε, N, C’, C_α, C_β chemical shifts (ppm) of apelin-55 with SDS micelles.

Residue	H_N	N	C’	C_α	C_β	H_δ	H_ε	Comments
**0Ser**	**-**	**-**	**171.46**	**57.72**	**63.11**	**-**	**-**	
**1Gly**	**8.62**	**109.76**	**173.92**	**45.63**	**-**	**-**	**-**	
2?Ser #1	8.21	115.30	-	58.72	-	-	-	No assignable C’ and C_β peak
2?Ser #2	8.16	114.98	174.30	58.60	64.12	-	-	-
**2Ser**	**8.17**	**115.20**	**174.36**	**58.68**	**64.04**	**-**	**-**	**-**
3?Leu #1	8.18	122.85	175.20	56.57	43.50	-	-	-
**3Leu**	**8.13**	**122.61**	**176.52**	**56.08**	**43.48**	**-**	**-**	**-**
4?Met #1	7.82	117.36	174.96	53.09	33.29	-	-	-
4?Met #2	7.36	113.38	177.20	52.47	33.97	-	-	-
**4Met**	**7.78**	**117.94**	**175.87**	**53.25**	**33.14**	**-**	**-**	**-**
5?Pro #1	-	-	175.70	62.98	-	-	-	Coming from 6?Leu #1<br>No assignable C_β peak
5?Pro #2	-	-	175.60	62.83	-	-	-	Coming from 6?Leu #2<br>No assignable C_β peak
**5Pro**	**-**	**-**	**175.58**	**62.83**	**31.18**	**-**	**-**	**Coming from 6Leu**
6?Leu #1	7.86	121.80	-	52.88	-	-	-	No assignable C’ and C_β peaks
6?Leu #2	7.84	121.27	-	52.96	-	-	-	No assignable C’ and C_β peaks
**6Leu**	**7.79**	**121.16**	**175.66**	**52.85**	**41.99**	**-**	**-**	**-**
7?Pro #1	-	-	176.21	63.07	-	-	-	Coming from 8?Asp #1<br>No assignable C_β peaks

**7Pro**	**-**	**-**	**176.38**	**63.35**	**31.91**	**-**	**-**	**Coming from 8Asp**
8?Asp #1	8.54	120.68	-	54.46	-	-	-	No assignable C' and C_β peaks
**8Asp**	**8.24**	**119.24**	**176.91**	**54.28**	**41.16**	**-**	**-**	**-**
9?Gly #1	8.26	109.11	174.47	45.77	-	-	-	-
9?Gly #2	8.41	108.37	174.43	45.80	-	-	-	-
9?Gly #3	8.15	109.06	173.34	45.52	-	-	-	-
**9Gly**	**8.22**	**108.97**	**174.45**	**45.8**	**-**	**-**	**-**	**-**
10?Asn #1	7.98	123.76	-	54.85	-	-	-	No assignable C' and C_β peak
10?Asn #2	7.95	123.76	-	54.82	40.81	-	-	No assignable C' peak
10?Asn #3	8.33	118.18	-	53.73	38.84	-	-	No assignable C' peak
**10Asn**	**8.26**	**118.2**	**175.74**	**53.75**	**39.28**	**6.83, 7.56**	**-**	**-**
**11Gly**	**8.4**	**108.61**	**174.38**	**45.76**	**-**	**-**	**-**	**-**
**12Leu**	**8.06**	**120.94**	**177.7**	**55.6**	**-**	**-**	**-**	**No assignable C_β peak**
13?Glu #1	8.21	119.49	176.41	57.04	29.54	-	-	-
13?Glu #2	8.38	120.39	176.21	56.82	29.54	-	-	-
**13Glu**	**8.25**	**119.83**	**176.38**	**57.03**	**29.60**	**-**	**-**	**-**
14?Asp #1	8.20	119.74	176.47	54.40	-	-	-	No assignable C_β peak
**14Asp**	**8.18**	**119.32**	**176.45**	**54.61**	**40.88**	**-**	**-**	**-**
15?Gly #1	8.18	108.52	174.23	45.77	-	-	-	-
**15Gly**	**8.13**	**108.34**	**174.29**	**45.89**	**-**	**-**	**-**	**-**
16?Asn #1	8.21	118.06	175.71	53.99	39.42	-	-	-
**16Asn**	**8.18**	**118.12**	**176.07**	**53.92**	**39.24**	**6.88, 7.54**	**-**	**-**

**17Val**	**8.15**	**119.73**	**176.48**	**64.47**	**-**	**-**	**-**	**No assignable C_β peak**
**18Arg**	**7.99**	**119.49**	**176.5**	**57.69**	**29.93**	**-**	**-**	**-**
19?His #1	8.02	116.10	-	55.96	-	-	-	No assignable C' and C_β peak
19?His #2	7.95	115.44	174.84	56.12	-	-	-	No assignable C_β peak
**19His**	**7.97**	**115.71**	**174.80**	**56.07**	**28.71**	**-**	**-**	**-**
**20Leu**	**7.95**	**120.22**	**176.81**	**56.35**	**-**	**-**	**-**	**-**
**21Val**	**7.38**	**114.53**	**174.74**	**61.74**	**32.65**	**-**	**-**	**-**
**22Gln**	**7.84**	**122.34**	**173.57**	**53.84**	**29.16**	**-**	**6.74, 7.39**	**-**
23?Pro #1	-	-	176.13	62.31	34.64	-	-	Coming from 24?Arg #1
**23Pro**	**-**	**-**	**177.28**	**63.44**	**32.21**	**-**	**-**	**Coming from 24Arg**
24?Arg #1	8.30	122.42	176.14	54.00	32.29	-	-	-
**24Arg**	**8.28**	**120.43**	**177.3**	**57.13**	**30.75**	**-**	**-**	**-**
**25Gly**	**8.33**	**109.24**	**174.46**	**45.67**	**-**	**-**	**-**	**-**
26?Ser #1	8.06	114.82	174.36	58.62	64.03	-	-	-
**26Ser**	**8.03**	**114.82**	**174.41**	**58.64**	**64.04**	**-**	**-**	**-**
27?Arg #1	8.02	121.32	175.84	56.16	-	-	-	No assignable C_β peak
27?Arg #2	8.17	122.06	176.02	56.18	30.81	-	-	-
**27Arg**	**8.05**	**121.65**	**175.99**	**56.32**	**30.66**	**-**	**-**	**-**
28?Asn #1	8.35	119.34	175.82	53.25	39.45	-	-	-
28?Asn #2	8.44	119.88	-	53.29	-	-	-	No assignable C' and C_β peak
**28Asn**	**8.40**	**119.53**	**175.04**	**53.34**	**39.47**	**6.80, 7.50**	**-**	**-**
29?Gly #1	7.98	108.50	171.84	44.70	-	-	-	-

29?Gly #2	7.98	108.50	171.84	44.44	-	-	-	-
**29Gly**	**7.94**	**108.80**	**171.59**	**44.70**	**-**	**-**	**-**	**-**
**30Pro**	**-**	**-**	**177.11**	**63.35**	**32.32**	**-**	**-**	**Coming from 31Gly**
**31Gly**	**8.05**	**108.4**	**172.78**	**44.65**	**-**	**-**	**-**	**-**
32?Pro #1	-	-	176.18	62.32	-	-	-	Coming from 33?Trp #1 No assignable C_β peak
32?Pro #2	-	-	176.76	63.92	-	-	-	Coming from 33?Trp #2 No assignable C_β peak
32?Pro #3	-	-	176.84	63.83	-	-	-	Coming from 33?Trp #3 No assignable C_β peak
**32Pro**	**-**	**-**	**176.81**	**63.84**	**31.9**	**-**	**-**	**Coming from 33Trp**
33?Trp #1	8.39	122.16	176.38	57.70	29.63	-	-	-
33?Trp #2	7.64	118.73	176.63	57.87	29.05	-	-	-
33?Trp #3	7.73	118.89	176.64	57.83	-	-	-	**No assignable C_β peak**
**33Trp**	**7.69**	**118.78**	**176.67**	**57.75**	**28.97**	**-**	**10.02**	**-**
34?Gln #1	8.25	122.34	176.30	55.93	29.51	-	-	-
**34Gln**	**7.92**	**120.58**	**176.68**	**56.52**	**29.63**	**-**	**6.74, 7.45**	**-**
35?Gly #1	7.82	108.05	174.79	45.62	-	-	-	-
**35Gly**	**7.78**	**108.05**	**174.79**	**45.65**	**-**	**-**	**-**	**-**
36?Gly #1	8.04	108.11	174.42	45.56	-	-	-	-
**36Gly**	**8.01**	**108.33**	**174.19**	**45.56**	**-**	**-**	**-**	**-**
37?Arg #1	8.04	119.28	176.43	56.63	29.79	-	-	-

37?Arg #2	8.18	121.35	174.49	55.59	-	-	-	No assignable C_β peak
**37Arg**	**7.98**	**119.35**	**176.36**	**56.53**	**29.79**	**-**	**-**	**-**
**38Arg**	**7.95**	**119.92**	**175.57**	**56.31**	**30.68**	**-**	**-**	**-**
**39Lys**	**7.91**	**119.81**	**176.21**	**56.19**	**33.2**	**-**	**-**	**-**
**40Phe**	**7.92**	**119.69**	**175.76**	**58.22**	**39.48**	**-**	**-**	**-**
**41Arg**	**8.01**	**120.1**	**176.09**	**56.52**	**29.83**	**-**	**-**	**-**
**42Arg**	**7.89**	**119.52**	**175.52**	**55.98**	**30.79**	**-**	**-**	**-**
**43Gln**	**8.09**	**119.00**	**175.34**	**55.77**	**29.76**	**-**	**6.74, 7.45**	**-**
**44Arg**	**8.07**	**121.27**	**174.41**	**54.03**	**29.81**	**-**	**-**	**-**
45?Pro #1	-	-	176.17	62.21	-	-	-	Coming from 46?Arg #1<br>No assignable C_β peak
45?Pro #2	-	-	176.46	62.44	-	-	-	Coming from 46?Arg #2<br>No assignable C_β peak
**45Pro**	**-**	**-**	**176.79**	**63.87**	**29.87**	**-**	**-**	**Coming from 46Arg**
46?Arg #1	8.60	121.59	-	56.73	-	-	-	No assignable C' or C_β peak
46?Arg #2	8.46	121.53	-	56.72	-	-	-	No assignable C' or C_β peak
**46Arg**	**8.13**	**117.94**	**176.46**	**56.48**	**30.7**	**-**	**-**	**-**
**47Leu**	**7.84**	**119.98**	**177.04**	**55.37**	**42.22**	**-**	**-**	**-**
**48Ser**	**7.92**	**113.84**	**174.54**	**58.73**	**63.99**	**-**	**-**	**-**
**49His**	**8.27**	**119.06**	**174.04**	**55.73**	**28.77**	**-**	**-**	**-**
50?Lys #1	8.06	120.26	176.07	56.12	-	-	-	No assignable C_β peak
**50Lys**	**8.08**	**120.86**	**176.37**	**56.29**	**33.26**	**-**	**-**	**-**

51?Gly #1	8.00	108.09	171.80	44.07	-	-	-	-
**51Gly**	**8.08**	**109.13**	**171.80**	**44.66**	**-**	**-**	**-**	**-**
52?Pro #1	-	-	175.78	63.36	-	-	-	Coming from 53?Met #1<br>No assignable C_β peak
52?Pro #2	-	-	176.22	63.45	29.30	-	-	Coming from 53?Met #2<br>No assignable C_β peak
52?Pro #3	-	-	176.30	62.38	34.80	-	-	Coming from 53?Met #3
52?Pro #4	-	-	176.57	62.23	-	-	-	Coming from 53?Met #4
**52Pro**	**-**	**-**	**176.62**	**63.20**	**-**	**-**	**-**	**Coming from 53Met**<br>**No assignable C_β peak**
53?Met #1	7.75	118.46	174.51	54.08	-	-	-	-
53?Met #2	8.15	118.84	174.04	54.25	31.83	-	-	-
53?Met #3	8.44	120.38	175.54	53.53	32.94	-	-	-
53?Met #4	8.11	120.09	177.14	53.36	33.01	-	-	-
**53Met**	**8.13**	**120.26**	**175.31**	**53.35**	**32.99**	**-**	**-**	**-**
54?Pro #1	-	-	174.82	63.50	28.20	-	-	Coming from 55?Phe #2
54?Pro #2	-	-	175.23	62.68	34.18	-	-	Coming from 55?Phe #2
**54Pro**	**-**	**-**	**175.07**	**63.48**	**31.29**	**-**	**-**	**Coming from 55Phe**

55?Phe #1	7.02	122.55	179.58	58.02	40.27	-	-	-
55?Phe #2	7.82	125.34	180.44	59.09	39.75	-	-	-
**55Phe**	**7.16**	**122.95**	**179.76**	**58.16**	**40.18**	**-**	**-**	**-**

Supplementary Table S4: H_N, H_δ, H_ε, N, C', C_α, C_β chemical shifts (ppm) of apelin-55 with LPPG micelles.

Residue	H_N	N	C'	C_α	C_β	H_δ	H_ε	Comments
**0Ser**	**-**	**-**	**-**	**-**	**-**	**-**	**-**	**-**
**1Gly**	**-**	**-**	**173.62**	**45.48**	**-**	**-**	**-**	**-**
**2Ser**	**8.24**	**115.34**	**174.39**	**58.31**	**64.16**	**-**	**-**	**-**
**3Leu**	**8.30**	**123.28**	**176.22**	**55.77**	**43.45**	**-**	**-**	**-**
**4Met**	**7.91**	**118.5**	**173.75**	**53.07**	**33.28**	**-**	**-**	**-**
5?Pro #1	-	-	176.01	62.91	-	-	-	Coming from 6?Leu #1
**5Pro**	**-**	**-**	**175.66**	**62.85**	**31.42**	**-**	**-**	**Coming from 6Leu**
6?Leu #1	8.06	122.69	-	52.74	-	-	-	No assignable C' and C_β peaks
**6Leu**	**7.95**	**121.74**	**175.51**	**52.69**	**42.20**	**-**	**-**	**-**
**7Pro**	**-**	**-**	**176.19**	**63.14**	**-**	**-**	**-**	**Coming from 8Asp**<br>**No assignable C_β peak**
**8Asp**	**8.28**	**119.53**	**176.87**	**54.18**	**41.29**	**-**	**-**	**-**
9?Gly #1	8.19	109.10	173.22	45.52	-	-	-	-
**9Gly**	**8.33**	**109.17**	**174.40**	**45.83**	**-**	**-**	**-**	**-**
10?Asn #1	7.95	123.95	-	54.89	40.82	-	-	No assignable C' peak
**10Asn**	**8.33**	**118.37**	**175.75**	**53.76**	**39.19**	**6.90, 7.61**	**-**	**-**
**11Gly**	**8.46**	**108.79**	**174.37**	**45.76**	**-**	**-**	**-**	**-**
**12Leu**	**8.08**	**120.93**	**177.49**	**55.54**	**42.21**	**-**	**-**	**-**
**13Glu**	**8.38**	**120.02**	**176.24**	**56.85**	**29.68**	**-**	**-**	**-**

14?Asp #1	8.16	119.86	176.66	54.50	41.06	-	-	-
**14Asp**	**8.21**	**120.61**	**176.66**	**54.50**	**41.08**	**-**	**-**	**-**
15?Gly #1	8.29	108.66	174.26	45.79	-	-	-	-
**15Gly**	**8.26**	**108.40**	**174.31**	**45.89**	**-**	**-**	**-**	**-**
**16Asn**	**8.27**	**118.65**	**176.08**	**54**	**39.05**	**6.86, 7.60**	**-**	**-**
17?Val #1	8.12	120.12	175.97	63.35	-	-	-	No assignable C_β peak
**17Val**	**8.17**	**119.94**	**176.21**	**63.98**	**32.06**	**-**	**-**	**-**
**18Arg**	**8.17**	**120.93**	**176.64**	**57.49**	**-**	**-**	**-**	**-**
**19His**	**8.11**	**116.59**	**-**	**55.85**	**-**	**-**	**-**	**No assignable C' and C_β peak**
**20Leu**	**-**	**-**	**176.59**	**56.54**	**-**	**-**	**-**	**Coming from 21Val**
**21Val**	**7.49**	**113.88**	**174.65**	**61.43**	**32.48**	**-**	**-**	**-**
**22Gln**	**7.93**	**122.35**	**173.64**	**53.6**	**29.4**	**-**	**6.80, 7.50***	**-**
**23Pro**	**-**	**-**	**176.77**	**63.33**	**-**	**-**	**-**	**Coming from 24Arg**
24?Arg #1	8.50	120.40	175.34	53.27	33.08	-	-	-
**24Arg**	**8.46**	**120.92**	**176.84**	**56.39**	**30.99**	**-**	**-**	**-**
**25Gly**	**8.42**	**109.19**	**174.09**	**45.44**	**-**	**-**	**-**	**-**
**26Ser**	**8.17**	**115.19**	**174.65**	**58.46**	**64.08**	**-**	**-**	**-**
27?Arg #1	8.39	122.56	175.90	56.10	30.77	-	-	-
**27Arg**	**8.36**	**122.30**	**175.92**	**56.33**	**30.69**	**-**	**-**	**-**
28?Asn #1	8.39	119.09	175.08	53.27	39.46	-	-	-
**28Asn**	**8.43**	**119.17**	**175.01**	**53.24**	**39.44**	**6.85, 7.57**	**-**	**-**
29?Gly #1	8.10	108.69	171.75	44.46	-	-	-	-

**29Gly**	**8.02**	**108.81**	**171.45**	**44.70**	**-**	**-**	**-**	**-**
30?Pro #1	-	-	177.16	63.20	-	-	-	Coming from 31?Gly #1<br>No assignable C_β peak
**30Pro**	**-**	**-**	**177.1**	**63.15**	**-**	**-**	**-**	**Coming from 31Gly**<br>**No assignable C_β peak**
31?Gly #1	8.10	108.27	-	44.60	-	-	-	No assignable C' peak
**31Gly**	**8.26**	**108.33**	**172.7**	**44.62**	**-**	**-**	**-**	**-**
**32Pro**	**-**	**-**	**176.55**	**63.96**	**-**	**-**	**-**	**Coming from 33Trp**<br>**No assignable C_β peak**
**33Trp**	**7.73**	**118.02**	**176.33**	**57.54**	**29.06**	**-**	**10.36**	**-**
**34Gln**	**7.98**	**120.55**	**176.9**	**56.93**	**29.06**	**-**	**6.81, 7.50***	**-**
**35Gly**	**8.12**	**108.35**	**175.03**	**45.89**	**-**	**-**	**-**	**-**
**36Gly**	**8.18**	**108.44**	**174.24**	**45.91**	**-**	**-**	**-**	**-**
37?Arg #1	8.22	121.47	-	55.45	-	-	-	No assignable C' or C_β peak
37?Arg #2	8.22	119.36	-	56.81	-	-	-	No assignable C' or C_β peak
**37Arg**	**8.15**	**119.57**	**176.65**	**57.29**	**30.66**	**-**	**-**	**-**
**38Arg**	**8.00**	**119.5**	**-**	**56.52**	**29.73**	**-**	**-**	**-**
**39Lys**	**-**	**-**	**176.1**	**56.34**	**-**	**-**	**-**	**Coming from 40Phe**<br>**No assignable C_β peak**
**40Phe**	**8.04**	**119.61**	**175**	**57.91**	**39.98**	**-**	**-**	**-**
**41Arg**	**8.01**	**121.33**	**175.57**	**55.93**	**31.3**	**-**	**-**	**-**
**42Arg**	**8.12**	**120.94**	**175.12**	**55.98**	**30.7**	**-**	**-**	**-**

**43Gln**	**7.92**	**120.18**	**174.71**	**55.16**	**31.15**	**-**	**6.83, 7.50***	**-**
**44Arg**	**8.50**	**122.17**	**174.00**	**54.26**	**30.57**	**-**	**-**	**-**
**45Pro**	**-**	**-**	**175.38**	**62.84**	**-**	**-**	**-**	**Coming from 46Arg**<br>**No assignable C_β peak**
**46Arg**	**8.29**	**120.72**	**175.58**	**55.58**	**31.36**	**-**	**-**	**-**
**47Leu**	**8.32**	**122.88**	**176.3**	**54.73**	**-**	**-**	**-**	**No assignable C_β peak**
**48Ser**	**8.10**	**115.16**	**174.11**	**58.09**	**64.28**	**-**	**-**	**-**
49?Lys #1	8.49	119.85	173.86	55.61	29.36	-	-	-
**49His**	**8.52**	**119.84**	**173.88**	**55.65**	**29.35**	**-**	**-**	**-**
**50Lys**	**8.32**	**122.29**	**176.25**	**56.19**	**33.38**	**-**	**-**	**-**
51?Gly #1	8.11	108.69	-	44.31	-	-	-	No assignable C’ peak
**51Gly**	**8.20**	**109.81**	**179.72**	**44.59**	**-**	**-**	**-**	**-**
**52Pro**	**-**	**-**	**176.4**	**63.09**	**-**	**-**	**-**	**Coming from 53Met**<br>**No assignable C_β peak**
**53Met**	**8.18**	**119.9**	**175.21**	**53.04**	**33.43**	**-**	**-**	**-**
54?Pro #1	-	-	174.57	63.31	-	-	-	Coming from 55?Phe #1<br>No assignable C_β peak
54?Pro #2	-	-	174.38	63.51	-	-	-	Coming from 55?Phe #2<br>No assignable C_β peak
**54Pro**	**-**	**-**	**174.59**	**63.42**	**31.19**	**-**	**-**	**Coming from 55Phe**
55?Phe #1	7.68	124.41	180.02	58.65	-	-	-	No assignable C_β peak
55?Phe #2	7.05	121.39	179.24	57.89	40.30	-	-	-

**55Phe**	**7.14**	**121.82**	**179.34**	**58.00**	**40.29**	**-**	**-**	**-**

*based on peak inference from assigned ^{1}H-^{15}N HSQC spectrum of apelin-55 in buffer at 37 °C

Supplementary Table S5: H_N and N chemical shifts (ppm) of apelin-55 with Brij-35 micelles.

Residue	H_N	N	Comments
**0Ser**	**-**	**-**	**-**
**1Gly**	**-**	**-**	**-**
**2Ser**	**8.27**	**115.77**	**-**
3?Leu #1	8.22	124.54	-
3?Leu #2	8.31	122.67	-
**3Leu**	**8.29**	**124.15**	**-**
4?Met #1	7.82	119.54	-
**4Met**	**8.20**	**122.25**	**-**
**5Pro**	**-**	**-**	**-**
6?Leu #1	7.93	121.54	-
6?Leu #2	8.39	123.09	-
**6Leu**	**8.22**	**123.43**	**-**
**7Pro**	**-**	**-**	**-**
8?Asp #1	8.25	119.31	-
**8Asp**	**8.25**	**119.91**	**-**
9?Gly #1	8.20	108.98	-
**9Gly**	**8.26**	**109.00**	**-**
10?Asn #1	7.94	124.06	-
**10Asn**	**8.37**	**118.59**	**-**
**11Gly**	**8.46**	**109.22**	**-**
12?Leu #1	8.17	121.36	-
**12Leu**	**8.05**	**121.22**	**-**
13?Glu #1	8.49	121.22	-
**13Glu**	**8.45**	**121.11**	**-**
**14Asp**	**8.24**	**121.25**	**-**
**15Gly**	**8.35**	**109.07**	**-**
**16Asn**	**8.31**	**118.71**	**-**
**17Val**	**7.91**	**119.3**	**-**

**18Arg**	**8.2**	**123.04**	**-**
**19His**	**-**	**-**	**-**
**20Leu**	**8.13**	**123.22**	**-**
21?Val #1	7.96	121.26	-
**21Val**	**8.04**	**120.99**	**-**
22?Gln #1	8.47	121.85	-
22?Gln #2	8.06	122.78	-
**22Gln**	**8.32**	**125.03**	**-**
**23Pro**	**-**	**-**	**-**
**24Arg**	**8.42**	**121.4**	**-**
**25Gly**	**8.39**	**110**	**-**
**26Ser**	**8.14**	**115.47**	**-**
**27Arg**	**8.38**	**122.57**	**-**
28?Asn #1	8.34	119.24	-
**28Asn**	**8.33**	**119.24**	**-**
**29Gly**	**8.1**	**109.23**	**-**
**30Pro**	**-**	**-**	**-**
31?Gly #1	8.07	109.51	-
**31Gly**	**8.14**	**108.84**	**-**
**32Pro**	**-**	**-**	**-**
33?Trp #1	8.07	120.66	-
**33Trp**	**8.02**	**120.50**	**-**
34?Gln #1	8.07	122.54	-
**34Gln**	**8.1**	**122.51**	**-**
**35Gly**	**7.67**	**108.63**	**-**
**36Gly**	**8.13**	**108.48**	**-**
**37Arg**	**8.14**	**120.37**	**-**
**38Arg**	**8.24**	**121.74**	**-**
**39Lys**	**8.16**	**122.15**	**-**
**40Phe**	**8.14**	**121.2**	**-**

**41Arg**	**-**	**-**	**-**
**42Arg**	**8.37**	**122.58**	**-**
**43Gln**	**8.41**	**122.12**	**-**
**44Arg**	**8.4**	**123.86**	**-**
**45Pro**	**-**	**-**	**-**
**46Arg**	**8.39**	**121.56**	**-**
**47Leu**	**8.23**	**123.48**	**-**
**48Ser**	**8.23**	**116.38**	**-**
**49His**	**-**	**-**	**-**
**50Lys**	**-**	**-**	**-**
51?Gly #1	8.08	110.36	-
51?Gly #2	8.17	109.29	-
51?Gly #3	8.18	109.64	-
**51Gly**	**8.14**	**110.39**	**-**
**52Pro**	**-**	**-**	**-**
53?Met #1	8.55	122.15	-
53?Met #2	8.03	119.30	-
**53Met**	**8.29**	**121.44**	**-**
**54Pro**	**-**	**-**	**-**
55?Phe #1	7.62	125.16	-
55?Phe #2	7.85	126.11	-
**55Phe**	**7.42**	**123.79**	**-**

Supplementary Table S6: H_N and N chemical shifts (ppm) of apelin-36 (main conformation only) in buffer, DPC, SDS, and LPPG micelles.

	Buffer		DPC		SDS		LPPG		
Residue	H_N	N	H_N	N	H_N	N	H_N	N	Comments
1Leu	8.12	123.18	-	-	-	-	-	-	No visible peak
2Val	-	-	-	-	-	-	-	-	No visible peak
3Gln	-	-	-	-	-	-	-	-	No visible peak
4Pro	-	-	-	-	-	-	-	-	No visible peak
5Arg	8.41	121.28	8.43	121.47	8.36	109.36	8.38	120.46	-
6Gly	8.39	110.03	8.4	109.98	8.24	109.02	8.41	109.1	-
7Ser	8.14	115.44	8.16	115.51	8.03	114.78	8.18	115.09	-
8Arg	8.4	122.62	8.39	122.64	8.08	121.67	8.4	122.26	-
9Asn	8.32	119.27	8.35	119.38	8.39	119.37	8.41	118.86	-
10Gly	8.11	109.26	8.21	108.76	7.94	108.82	8.02	108.77	-
11Pro	-	-	-	-	-	-	-	-	-
12Gly	8.15	108.82	8.21	108.76	8.05	108.45	8.24	108.18	-
13Pro			-	-	-	-	-	-	-
14Trp	8.03	120.59	8.06	119.81	7.69	118.88	7.68	117.96	-
15Gln	8.1	122.63	8.06	121.53	7.92	120.81	7.97	120.62	-
16Gly	7.64	108.58	7.87	108.53	7.78	108.12	8.13	108.39	-
17Gly	8.15	108.48	8.17	108.49	8.02	108.39	8.18	108.45	-

18Arg	8.16	120.39	8.2	120.25	7.99	119.39	8.16	119.66	-
19Arg	8.27	121.87	8.19	120.84	7.95	119.91	7.99	119.43	-
20Lys	8.17	122.17	-	-	7.91	119.89	-	-	No inferable peak in apelin-55
21Phe	8.15	121.22	8.13	120.4	7.93	119.76	8.04	119.69	-
22Arg	8.22	123.31	8.14	121.84	8.02	120.21	8.02	121.45	-
23Arg	8.35	122.69	8.25	121.66	7.88	119.58	8.13	121	-
24Gln	8.42	122.08	8.3	121.15	8.09	119.09	7.90	120.25	-
25Arg	8.4	123.88	8.39	123.25	8.08	121.34	8.53	122.25	-
26Pro	-	-	-	-	-	-	-	-	-
27Arg	8.4	121.55	8.39	121.11	8.13	118.03	8.29	120.78	-
28Leu	8.25	123.5	8.27	123.01	7.85	120.1	8.30	123.08	-
29Ser	8.23	116.32	8.2	115.85	7.93	113.95	8.10	115.24	-
30His	-	-	-	-	8.26	119.11	8.53	119.86	No inferable peak in apelin-55
31Lys	-	-	-	-	8.08	120.89	8.33	122.30	No inferable peak in apelin-55
32Gly	8.12	110.27	8.09	110.04	8.08	109.18	8.20	109.79	-
33Pro	-	-	-	-	-	-	-	-	-
34Met	8.3	121.42	8.03	119.26	8.13	120.31	8.18	119.94	-
35Pro	-	-	-	-	-	-	-	-	-

36Phe	7.43	123.79	7.31	123.13	7.16	122.74	7.15	121.84	-

Supplementary Table S7: NMR spectroscopy experimental parameters.

Experiment	Pulse program (Bruker)	Delay (s)	# of scans	Acquisition time (s)	# of complex points	Sweep width (ppm)	Center position (ppm)	^{1}H frequency (MHz)	Note
**Apelin-55 in DPC at 37°C**									**Backbone assignment**
HNCO	hncogp3d	1	8	^{1}H: 0.1216512	^{1}H: 2048	^{1}H: 12.0224	^{1}H: 4.705	700	
				^{15}N: 0.0182837	^{15}N: 48	^{15}N: 18.5000	^{15}N: 117.250		
				^{13}C: 0.0227165	^{13}C: 48	^{13}C: 6.0000	^{13}C: 173.000		
HN(CA)CO	hncacogp3d	1	16	^{1}H: 0.1216512	^{1}H: 2048	^{1}H: 12.0224	^{1}H: 4.705	700	
				^{15}N: 0.0182837	^{15}N: 48	^{15}N: 18.5000	^{15}N: 117.250		
				^{13}C: 0.0227165	^{13}C: 48	^{13}C: 6.0000	^{13}C: 173.000		
HNCA	hncagp3d	1	16	^{1}H: 0.1216512	^{1}H: 2048	^{1}H: 12.0224	^{1}H: 4.705	700	
				^{15}N: 0.0182837	^{15}N: 48	^{15}N: 18.5000	^{15}N: 117.250		
				^{13}C: 0.0126805	^{13}C: 96	^{13}C: 21.5000	^{13}C: 51.250		
HN(CO)CA	hncocagp3d	1	8	^{1}H: 0.1216512	^{1}H: 2048	^{1}H: 12.0224	^{1}H: 4.705	700	
				^{15}N: 0.0182837	^{15}N: 48	^{15}N: 18.5000	^{15}N: 117.250		
				^{13}C: 0.0126805	^{13}C: 48	^{13}C: 21.5000	^{13}C: 51.250		
HNCACB	hncacbgp3d	1	16	^{1}H: 0.1216512	^{1}H: 2048	^{1}H: 12.0224	^{1}H: 4.705	700	
				^{15}N: 0.0182837	^{15}N: 48	^{15}N: 18.5000	^{15}N: 117.250		
				^{13}C: 0.0086550	^{13}C: 128	^{13}C: 42.0000	^{13}C: 43.500		

^{1}H-^{15}N HSQC	hsqcetf3gpsi2	1	8	^{1}H: 0.1044480<br>^{15}N: 0.0450997	^{1}H: 2048<br>^{15}N: 128	^{1}H: 14.0025<br>^{15}N: 20.0000	^{1}H: 4.705<br>^{15}N: 117.000	700	
^{1}H-^{15}N Het. NOE HSQC	hsqcnoef3gpsi	5	32	^{1}H: 0.1216512<br>^{15}N: 0.1066547	^{1}H: 2048<br>^{15}N: 280	^{1}H: 12.0224<br>^{15}N: 18.5000	^{1}H: 4.705<br>^{15}N: 117.250	700	
**Apelin-55 in SDS at 37°C**									**Backbone assignment**
HNCO	hncogp3d	1	8	^{1}H: 0.1216512<br>^{15}N: 0.0180399<br>^{13}C: 0.0136299	^{1}H: 2048<br>^{15}N: 48<br>^{13}C: 48	^{1}H: 12.0224<br>^{15}N: 18.7500<br>^{13}C: 10.0000	^{1}H: 4.707<br>^{15}N: 116.350<br>^{13}C: 174.500	700	
HN(CA)CO	hncacogp3d	1	16	^{1}H: 0.1216512<br>^{15}N: 0.0180399<br>^{13}C: 0.0136299	^{1}H: 2048<br>^{15}N: 48<br>^{13}C: 48	^{1}H: 12.0224<br>^{15}N: 18.7500<br>^{13}C: 10.0000	^{1}H: 4.707<br>^{15}N: 116.350<br>^{13}C: 174.500	700	
HNCA	hncagp3d	1	16	^{1}H: 0.1216512<br>^{15}N: 0.0180399<br>^{13}C: 0.0054526	^{1}H: 2048<br>^{15}N: 48<br>^{13}C: 48	^{1}H: 12.0224<br>^{15}N: 18.7500<br>^{13}C: 25.0000	^{1}H: 4.707<br>^{15}N: 116.350<br>^{13}C: 51.000	700	
HN(CO)CA	hncocagp3d	1	16	^{1}H: 0.1216512<br>^{15}N: 0.0180399<br>^{13}C: 0.0054526	^{1}H: 2048<br>^{15}N: 48<br>^{13}C: 48	^{1}H: 12.0224<br>^{15}N: 18.7500<br>^{13}C: 25.0000	^{1}H: 4.707<br>^{15}N: 116.350<br>^{13}C: 51.000	700	
HNCACB	hncacbgp3d	1	16	^{1}H: 0.1216512<br>^{15}N: 0.0180399<br>^{13}C: 0.0070683	^{1}H: 2048<br>^{15}N: 48<br>^{13}C: 112	^{1}H: 12.0224<br>^{15}N: 18.7500<br>^{13}C: 45.0000	^{1}H: 4.707<br>^{15}N: 116.350<br>^{13}C: 40.500	700	

^{1}H-^{15}N HSQC	hsqcetf3gpsi2	1	16	^{1}H: 0.1216512	^{1}H: 2048	^{1}H: 12.0224	^{1}H: 4.707	700	
				^{15}N: 0.0481064	^{15}N: 128	^{15}N: 18.7500	^{15}N: 116.350		
^{1}H-^{15}N Het. NOE HSQC	hsqcnoef3gpsi	5	32	^{1}H: 0.1044480	^{1}H: 2048	^{1}H: 14.0025	^{1}H: 4.699	700	
				^{15}N: 0.1080234	^{15}N: 256	^{15}N: 16.7000	^{15}N: 115.650		
**Apelin-55 in LPPG at 37°C**									**Backbone assignment**
HNCO	hncogp3d	1	8	^{1}H: 0.1216512	^{1}H: 2048	^{1}H: 12.0224	^{1}H: 4.705	700	
				^{15}N: 0.0187916	^{15}N: 48	^{15}N: 18.0000	^{15}N: 116.750		
				^{13}C: 0.0170374	^{13}C: 48	^{13}C: 8.0000	^{13}C: 174.000		
HN(CA)CO	hncacogp3d	1	16	^{1}H: 0.1216512	^{1}H: 2048	^{1}H: 12.0224	^{1}H: 4.705	700	
				^{15}N: 0.0187916	^{15}N: 48	^{15}N: 18.0000	^{15}N: 116.750		
				^{13}C: 0.0170374	^{13}C: 48	^{13}C: 8.0000	^{13}C: 174.000		
HNCA	hncagp3d	1	16	^{1}H: 0.1216512	^{1}H: 2048	^{1}H: 12.0224	^{1}H: 4.705	700	
				^{15}N: 0.0187916	^{15}N: 48	^{15}N: 18.0000	^{15}N: 116.750		
				^{13}C: 0.0079024	^{13}C: 48	^{13}C: 23.0000	^{13}C: 51.000		
HN(CO)CA	hncocagp3d	1	16	^{1}H: 0.1216512	^{1}H: 2048	^{1}H: 12.0224	^{1}H: 4.705	700	
				^{15}N: 0.0187916	^{15}N: 48	^{15}N: 18.0000	^{15}N: 116.750		
				^{13}C: 0.0079024	^{13}C: 64	^{13}C: 23.0000	^{13}C: 51.000		
HNCACB	hncacbgp3d	1	16	^{1}H: 0.1216512	^{1}H: 2048	^{1}H: 12.0224	^{1}H: 4.705	700	
				^{15}N: 0.0187916	^{15}N: 48	^{15}N: 18.0000	^{15}N: 116.750		
				^{13}C: 0.0080781	^{13}C: 128	^{13}C: 45.0000	^{13}C: 40.500		

^{1}H-^{15}N HSQC	hsqcetf3gpsi2	1	16	^{1}H: 0.1044480<br>^{15}N: 0.0450997	^{1}H: 2048<br>^{15}N: 128	^{1}H: 14.0025<br>^{15}N: 20.0000	^{1}H: 4.705<br>^{15}N: 116.750	700	
^{1}H-^{15}N Het. NOE HSQC	hsqcnoef3gpsi	5	32	^{1}H: 0.1216512<br>^{15}N: 0.0939578	^{1}H: 2048<br>^{15}N: 240	^{1}H: 12.0224<br>^{15}N: 18.0000	^{1}H: 4.707<br>^{15}N: 116.750	700	
**Apelin-55 in buffer at 37°C**									**^{15}N-labeled/ Diffusion**
^{1}H DOSY	ledbpgppr2s	1	128	^{1}H: 0.8579424	^{1}H: 12016<br>16 points	^{1}H: 14.0019	^{1}H: 4.706	500	
^{15}N-DOSY	led1dhsqc2d.jkr	1	2560	^{1}H: 0.1462272	^{1}H: 2048<br>9 points	^{1}H: 14.0019	^{1}H: 4.706	500	
^{1}H-^{15}N HSQC	hsqcetfpgpsi2	1	64	^{1}H: 0.1462272<br>^{15}N: 0.0507348	^{1}H: 2048<br>^{15}N: 144	^{1}H: 14.0019<br>^{15}N: 28.0000	^{1}H: 4.706<br>^{15}N: -261.500	500	
**Apelin-55 in Brij-35 at 37°C**									**^{15}N-labeled/ Diffusion**
^{1}H-DOSY	ledbpgppr2s	1	32	^{1}H: 0.8579424	^{1}H: 12016<br>16 points	^{1}H: 14.0019	^{1}H: 4.705	500	
^{1}H-DOSY (water)	ledbpgp2s	1	8	^{1}H: 0.9997428	^{1}H: 14002<br>16 points	^{1}H: 14.0019	^{1}H: 4.706	500	
^{15}N-DOSY	led1dhsqc2d.jkr	1	2560	^{1}H: 0.1462272	^{1}H: 2048<br>13 points	^{1}H: 14.0019	^{1}H: 4.705	500	
^{1}H-^{15}N HSQC	hsqcetfpgpsi2	1	64	^{1}H: 0.1462272	^{1}H: 2048	^{1}H: 14.0019	^{1}H: 4.705	500	

				^{15}N: 0.0507347	^{15}N: 144	^{15}N: 28.0000	^{15}N: -261.000		
**Apelin-55 in DPC at 37°C**									**^{15}N-labeled/ Diffusion**
^{1}H-DOSY	ledbpgppr2s	1	32	^{1}H: 0.8579424	^{1}H: 12016<br>16 points	^{1}H: 14.0019	^{1}H: 4.705	500	
^{15}N-DOSY	led1dhsqc2d.jkr	1	2560	^{1}H: 0.1462272	^{1}H: 2048<br>9 points	^{1}H: 14.0019	^{1}H: 4.705	500	
^{1}H-^{15}N HSQC	hsqcetfpgpsi2	1	64	^{1}H: 0.1462272<br>^{15}N: 0.0507348	^{1}H: 2048<br>^{15}N: 144	^{1}H: 14.0019<br>^{15}N: 28.0000	^{1}H: 4.705<br>^{15}N: -261.500	500	
**Apelin-55 in LPPG at 37°C**									**^{15}N-labeled/ Diffusion**
^{1}H-DOSY	ledbpgppr2s	1	32	^{1}H: 0.8579424	^{1}H: 12016<br>16 points	^{1}H: 14.0019	^{1}H: 4.705	500	
^{15}N-DOSY	led1dhsqc2d.jkr	1	2560	^{1}H: 0.1462272	^{1}H: 2048<br>9 points	^{1}H: 14.0019	^{1}H: 4.705	500	
^{1}H-^{15}N HSQC	hsqcetfpgpsi2	1	64	^{1}H: 0.1462272<br>^{15}N: 0.0507348	^{1}H: 2048<br>^{15}N: 144	^{1}H: 14.0019<br>^{15}N: 28.0000	^{1}H: 4.705<br>^{15}N: -261.500	500	
**Apelin-55 in SDS at 37°C**									**^{15}N-labeled/ Diffusion**
^{1}H-DOSY	ledbpgppr2s	1	32	^{1}H: 0.8579424	^{1}H: 12016<br>16 points	^{1}H: 14.0019	^{1}H: 4.705	500	

^{15}N-DOSY	led1dhsqc2d.jkr	1	2560	^{1}H: 0.1462272	^{1}H: 2048<br>9 points	^{1}H: 14.0019	^{1}H: 4.705	500	
^{1}H-^{15}N HSQC	hsqcetfpgpsi2	1	64	^{1}H: 0.1462272<br>^{15}N: 0.0507348	^{1}H: 2048<br>^{15}N: 144	^{1}H: 14.0019<br>^{15}N: 28.0000	^{1}H: 4.705<br>^{15}N: -261.500	500	
**Apelin-36 in buffer at 37°C**									**^{15}N-labeled/ Diffusion**
^{15}N-DOSY	led1dhsqc2d.jkr	1	2560	^{1}H: 0.1703936	^{1}H: 2048<br>13 points	^{1}H: 12.0160	^{1}H: 4.706	500	
^{1}H-^{15}N HSQC	hsqcetfpgpsi2	1	64	^{1}H: 0.1703936<br>^{15}N: 0.0526138	^{1}H: 2048<br>^{15}N: 160	^{1}H: 12.0160<br>^{15}N: 30.0000	^{1}H: 4.706<br>^{15}N: -261.500	500	
**Apelin-36 in DPC at 37°C**									**^{15}N-labeled/ Diffusion**
^{15}N-DOSY	led1dhsqc2d.jkr	1	2560	^{1}H: 0.1703936	^{1}H: 2048<br>13 points	^{1}H: 12.0160	^{1}H: 4.706	500	
^{1}H-^{15}N HSQC	hsqcetfpgpsi2	1	64	^{1}H: 0.1703936<br>^{15}N: 0.0526138	^{1}H: 2048<br>^{15}N: 160	^{1}H: 12.0160<br>^{15}N: 30.0000	^{1}H: 4.706<br>^{15}N: -261.500	500	
**Apelin-36 in LPPG at 37°C**									**^{15}N-labeled/ Diffusion**
^{15}N-DOSY	led1dhsqc2d.jkr	1	2560	^{1}H: 0.1703936	^{1}H: 2048<br>13 points	^{1}H: 12.0160	^{1}H: 4.707	500	
^{1}H-^{15}N HSQC	hsqcetfpgpsi2	1	64	^{1}H: 0.1703936	^{1}H: 2048<br>^{15}N: 160	^{1}H: 12.0160	^{1}H: 4.707	500	

				^{15}N: 0.0526138		^{15}N: 30.0000	^{15}N: -261.500		
**Apelin-36 in SDS at 37°C**									**^{15}N-labeled/ Diffusion**
^{15}N-DOSY	led1dhsqc2d.jkr	1	2560	^{1}H: 0.1703936	^{1}H: 2048<br>13 points	^{1}H: 12.0160	^{1}H: 4.708	500	
^{1}H-^{15}N HSQC	hsqcetfpgpsi2	1	64	^{1}H: 0.1703936<br>^{15}N: 0.0526138	^{1}H: 2048<br>^{15}N: 160	^{1}H: 12.0160<br>^{15}N: 30.0000	^{1}H: 4.708<br>^{15}N: -261.500	500	
**Buffer at 37°C**									**^{15}N-labeled/ Diffusion**
^{1}H-DOSY (water)	ledbpgp2s	1	8	^{1}H: 0.9997312	^{1}H: 12016<br>16 points	^{1}H: 12.0160	^{1}H: 4.706	500	
**Brij-35 at 37°C**									**^{15}N-labeled/ Diffusion**
^{1}H-DOSY	ledbpgppr2s	1	32	^{1}H: 0.8579424	^{1}H: 12016<br>16 points	^{1}H: 14.0019	^{1}H: 4.706	500	
^{1}H-DOSY (water)	ledbpgp2s	1	8	^{1}H: 0.9997428	^{1}H: 14002<br>16 points	^{1}H: 14.0019	^{1}H: 4.706	500	
**DPC at 37°C**									**^{15}N-labeled/ Diffusion**
^{31}P DOSY	ledbpgp2s	1	16	^{31}P: 1.0223616	^{31}P: 16384<br>16 points	^{31}P: 39.5780	^{31}P: 0.00	500	

**LPPG at 37°C**									**^{15}N-labeled/ Diffusion**
^{31}P DOSY	ledbpgp2s	1	16	^{31}P: 1.0223616	^{31}P: 16384<br>16 points	^{31}P: 39.5780	^{31}P: 0.00	500	
**SDS at 37°C**									**^{15}N-labeled/ Diffusion**
^{1}H DOSY	ledbpgppr2s	1	32	^{1}H: 0.8579424	^{1}H: 12016<br>16 points	^{1}H: 14.0019	^{1}H: 4.705	500	

Reference

1 Shin, K. *et al.* Bioactivity of the putative apelin proprotein expands the repertoire of apelin receptor ligands. *Biochim Biophys Acta* **1861**, 1901-1912, doi:10.1016/j.bbagen.2017.05.017 (2017).
